# High- and low-field NMR in binary solvent gradients

**DOI:** 10.1039/d6an00031b

**Published:** 2026-03-20

**Authors:** Haider Hussain, Paulina Putko, Dariusz Gołowicz, Krzysztof Kazimierczuk, Matthew Wallace

**Affiliations:** a School of Chemistry, Pharmacy and Pharmacology, University of East Anglia Norwich Research Park Norwich NR4 7TJ UK Matthew.Wallace@uea.ac.uk; b Centre of New Technologies, University of Warsaw Banacha 2C Warsaw 02-097 Poland k.kazimierczuk@cent.uw.edu.pl; c Institute of Physical Chemistry, Polish Academy of Sciences Kasprzaka 44/52 Warsaw 01-224 Poland

## Abstract

We introduce composition gradients of the solvent as a powerful new dimension for NMR analysis on both high-field and benchtop instruments. Taking advantage of the differences in the density and miscibility of binary solvent mixtures with different compositions, we layer two solutions at opposite extremes of the compositional range in an NMR tube. The diffusion of the layers into each other establishes a continuous variation in the solvent composition across the sample. Spatially resolved analysis of the sample using either chemical shift imaging (CSI) on high-field NMR instruments or physical movement of the sample (benchtop instruments) enables analysis of chemical systems as a function of the solvent composition. In high field, we determine the p*K*_a_ of poorly water-soluble active pharmaceutical ingredients (APIs) in a wide range of compositions of dimethylsulfoxide (DMSO)/water and perform accurate extrapolations to aqueous p*K*_a_ using the Yasuda-Shedlovsky method. We thus condense hours of tedious experiments, where the p*K*_a_ would be determined separately at each solvent composition, into a single 20 minute experiment. We can also detect the minimum quantity of DMSO required to maintain an API in the freely dissolved state. On a benchtop instrument, we demonstrate how our approach enables the transfer of resonance assignments between spectra of the same compound (asarone) acquired in different solvents (methanol and DMSO). We also show that the method can boost the spectral resolution of complex molecular mixtures (naproxen tablet) *via* the differential solubility of the components in the two solvents.

## Abstract 2

Gradients in the solvent composition of NMR samples are generated by layering two solvents of contrasting densities. Spectra are recorded at different compositions by physical movement of the tube (low-field) or chemical shift imaging (high-field).

## Graphical abstract

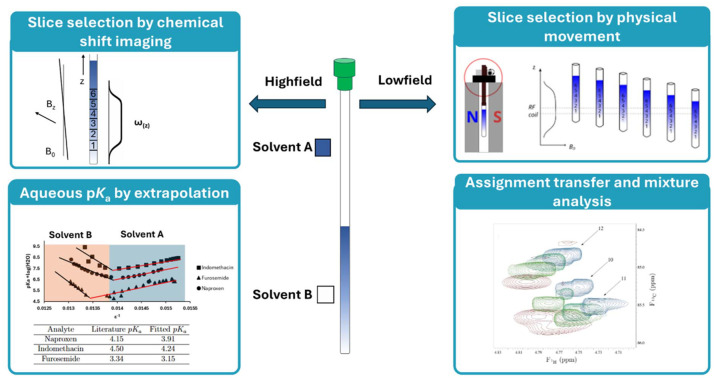

## 1 Introduction

The solvent in which a chemical system is present is a fundamental component that must be considered to understand the dynamics and kinetics of many chemical systems.^1^ A solvent is commonly understood as a part of a solution containing multiple components, where for convenience, one of the components is treated separately and labeled as the solvent. The solvent itself may be a mixture of compounds; nevertheless, the whole mixture can be treated separately from the other components and be labeled a solvent and the rest as solutes.^2,3^

From the definition of what a solvent is alone one can grasp the relative importance of it with respect to the chemical systems present within it given that the solvent is typically the chemical present in the highest quantity, surrounds all solutes in a way that maximizes solvent–solute interactions, and is the medium in which the solutes “swim”. The solvent thus impacts all the exchange dynamics that occur between the different solutes, such as molecular diffusion, ion transport, and heat transport to and from chemical reactions.^2,4,5^ These properties have a significant impact on the solubility, stability and reaction rates of chemical systems.^6–11^

Many studies have been conducted to determine the impact of solvent effects on critical parameters such as p*K*_a_, ion binding and dissolution.^12,13^ These studies generally use a binary solvent mixture where the solvent composition is changed. The effect that this change has on the parameter of interest is measured. For example, potentiometric titrations of pharmaceutically relevant compounds in DMSO/H_2_O solution mixtures have been performed to determine the aqueous p*K*_a_ of compounds that are too insoluble in water for them to be measured directly.^14^ Additionally, solvent effects are used for distinguishing chemical compounds *via* the differing reactions of these compounds to changes in solvent composition.^15–18^ However, a limitation of these approaches is the need for successive manual alteration of the solvent composition, which can have a significant time and material cost associated with it.

In this paper, we present a method of establishing a gradient in solvent composition across the NMR tube due to solvent diffusion. Multiple spatially resolved NMR spectra are then obtained, which enable chemical systems to be analysed as a function of solvent composition in a “single-shot” NMR experiment. This approach is implemented in a high-field spectrometer to determine the aqueous p*K*_a_ of water-insoluble compounds in a 20 minute NMR experiment. A similar approach, exploiting the vertical shift of a sample, is also implemented in two exemplar applications on benchtop instruments. In the first, the resonance assignments of asarone are transferred between methanol and DMSO. In the second, the complex mixtures are resolved *via* the difference in solubility of the different components of the mixture in the two solvents. Continued advances in the resolution and sensitivity of benchtop instruments, along with the provision of pulsed field gradient coils along the vertical axis of the sample tube, may enable the high-field experiments to be implemented on benchtop spectrometers.

## 2 Methods

### 2.1 Materials

All chemicals were purchased from Fisher Scientific or Sigma-Aldrich and used as received. Commercially available naproxen tablets were manufactured by Aflofarm Farmacja Polska Sp. z o.o. (Poland).

2,2-Dimethyl-2-silapentane-5-sulfonate (DSS) was the chemical shift reference used in all experiments at high-field. The proton transfer indicators used to determine p*K*_a_ as a function of solvent composition were 1,2,4-triazole, sodium formate, sodium acetate, 2-methylimidazole and sodium glycolate. Sodium methanesulfonate (NaMSA) was used as a solvent composition indicator. Stocks of these indicators in 20% H_2_O and 80% DMSO-d_6_, 80% H_2_O and 20% DMSO-d_6_ fractional volume (*f*) were prepared and used throughout the study. The analytes studied at high-field were naproxen, indomethacin, furosemide, quinine hydrochloride, salicylic acid, acetic acid, formic acid, sulfoacetic acid and glycolic acid. Stock solutions of the analytes in H_2_O were prepared at 10 mM concentration of water soluble compounds and 5 mM for sparingly soluble compounds.

For low-field experiments, stock solutions of α-asarone were prepared at 300.0 mM concentration in 0.5 ml DMSO-d_6_ and 2.0 ml deuterated methanol (MeOD). Naproxen tablets require prior preparation; they were crushed in a mortar and prepared to obtain *ca.* 10 mM concentration of naproxen in two samples: 100% DMSO-d_6_ and a mixture of 20% DMSO-d_6_ and 80% D_2_O. According to the producer, each tablet (*ca.* 280 mg) contains 200 mg naproxen and excipients: 38.9 mg of lactose monohydrate and undeclared amounts of corn starch, magnesium stearate, polyvidone, colloidal silica, sodium dodecyl sulfate, and sunset yellow (E 110). All samples were shaken for 5 minutes on a vortex mixer, then the supernatant was collected for analysis (the precipitate contained probably only colloidal silica and some amount of starch).

### 2.2 NMR

The experiments to determine p*K*_a_ by Yasuda-Shedlovsky extrapolation were performed on a Bruker Avance III 500 MHz spectrometer operating at 500.21 MHz for ^1^H equipped with a Bruker SampleXpress sample changer that was used for repeated injection and reinsertion of samples (Fig. S6). Locking and shimming was performed on DMSO-d_6_. The probe was equipped with *z*-axis pulsed field gradients. The temperature of the samples was maintained at 298 ± 0.5 K. The 90° pulse was 10 μs. The relaxation delay was 3 seconds. The spectral width was 20 ppm, and the number of data points were 32k, giving an acquisition time of 2.15 seconds. CSI experiments were performed using a gradient phase encoding sequence incorporating perfect echo excitation sculpting (ES) for water suppression.^19,20^ A spoil gradient (27 G cm^−1^) was employed at the end of the signal acquisition period to destroy any transverse magnetization. The gradient pulse was 166 μs in duration and varied between −8 and 8 G cm^−1^ in 32 steps. The shape of the pulse was a smoothed square. 4 ms Gaussian 180° pulses were employed for inverting magnetisation of the H_2_O region. Four scans were acquired at each step giving a total acquisition time of 23 min. Sixteen dummy scans were acquired prior to signal acquisition. DSS was used as a chemical shift reference in all experiments (0 ppm). NMR data was processed in Bruker TopSpin 4.6.5 and Mnova 14.2.0 and CSI images were processed in phase sensitive mode following the procedure of Trigo-Mourino *et al.*^19^ Following two-dimensional Fourier transformation of the CSI data sets, the individual spectra were automatically extracted, any residual phase errors corrected, and the spectra referenced to DSS (0 ppm) using an automation script written in house.^20^ Analyte and indicator chemical shift data were exported from TopSpin and Mnova into Microsoft Excel and subsequently analysed.

Experiments at low-field were performed on a Spinsolve Carbon 43 MHz benchtop spectrometer (Magritek GmbH, Germany). We used the SWAPE device^21^ to change the vertical position of the sample mechanically. All samples were measured at 16 different positions starting from 2 cm above the bottom of the NMR tube, and each successive value of the sample represents a step of 0.5 cm, while the active region of the RF coil is 1 cm. We performed ^1^H NMR and ^1^H–^13^C HSQC experiments for each volume of the asarone sample and ^1^H NMR experiment for the naproxen sample. The experimental time for a single volume was 62 minutes for asarone and 4 minutes for naproxen. ^1^H spectra were acquired with 8192 data points, giving an acquisition time of 1.64 seconds. The relaxation delay was 1.86 seconds, and 32 (asarone) or 64 (naproxen tablet) transients. The pulse length was 11.5 μs. Both ^1^H and ^1^H–^13^C HSQC experiment were set up using Spinsolve Expert software (version 2.01.19). The ^1^H and ^13^C pulses were 61.1 μs. The non-uniform sampling (NUS) has been used (64 random points out of a full grid of 1024 increments). The NUS reconstruction was performed using a compressed sensing module of mddnmr software.^22^ The iteratively re-weighted least squares algorithm^23^ has been used with 20 iterations and a virtual echo option.^24^

### 2.3 Establishing solvent gradients for NMR analysis

**Fig. 1:**
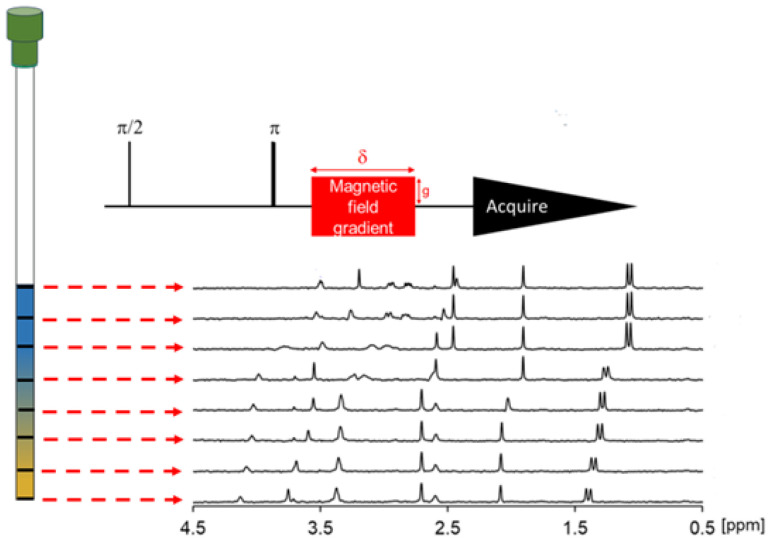
Scheme of solvent gradient experiment. 0.2*f*_DMSO_ (blue) is layered on top of 0.8*f*_DMSO_. A gradient is formed, leading to changes in chemical shift when analysed by chemical shift imaging.

To establish a solvent gradient spanning between 0.8*f*_DMSO_ and 0.2*f*_DMSO_ in a 5 mm NMR tube for analysis at high-field (Fig. 1), two solutions were prepared at both of these compositions containing the analyte, DSS, NaMSA, and the indicator/indicators. The concentration of the analyte was equal to the concentration of the indicator when one indicator was used. When multiple indicators were used (furosemide), the sum of their concentration equalled the concentration of the analyte (section S9). 320 μl of the 0.8*f*_DMSO_ solution was put into the NMR tube. This was followed by 320 μl of the 0.2*f*_DMSO_ solution being gently layered on top using a 9 inch Pasteur pipette. The 0.8*f*_DMSO_ solution was put at the bottom of the NMR tube due to it having a higher density. 2D ^1^H chemical shift imaging experiments were then recorded on the sample. A smooth variation in solvent composition across the full range of compositions was obtained approximately 4 hours after preparation of the sample (see SI, section S8 for how that is determined). We note that a similar approach can be used to generate a smooth variation in the solvent composition between DMSO and chloroform (SI, section S12).

**Fig. 2:**
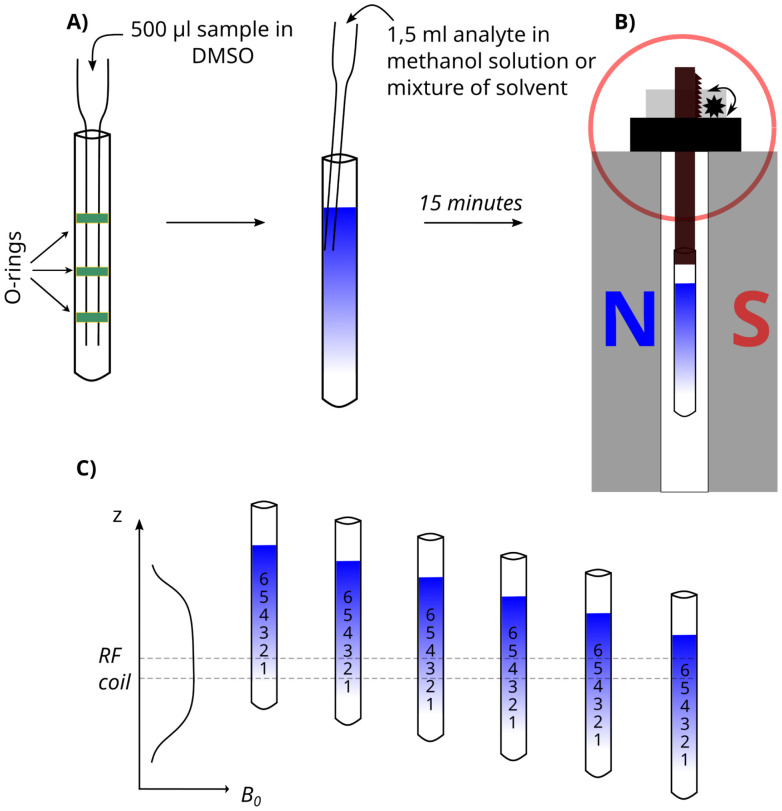
Scheme of a solvent-gradient experiment using a 43 MHz BT-NMR. (A) General sample preparation procedure (the o-rings serve to guide the pipette down the centre of the sample tube). (B) Sample in SWAPE device (SWAPE motor marked in red) inside BT-NMR spectrometer. (C) The scheme of vertical sample shifting with respect to a *B*_0_ field profile.

Samples for analysis at low-field were prepared using thin-walled NMR tubes 9″ in length and 5 mm in outer diameter. The first step to establish a solvent gradient was to place a sample dissolved in DMSO-d_6_ at the bottom of the NMR tube using a glass pipette (see Fig. 2). Then, we froze the sample in the refrigerator at a temperature of 6 °C for about 15 minutes. Freezing the DMSO sample helps reduce solvent mixing during sample preparation. Then, we added a solution containing the studied substance and a second solvent – MeOD for asarone or a mixture of deuterium oxide and DMSO for naproxen tablet.

## 3 Results and discussion

### 3.1 Determination of p*K*_a_ of analyte in pure water by Yasuda-Shedlovsky extrapolation

The Yasuda-Shedlovsky extrapolation equation is an empirical equation that linearly correlates the p*K*_a_ of a compound in a particular solvent composition with the dielectric constant (eqn (1)). It is a robust method for predicting the aqueous p*K*_a_ values of water-insoluble system by extrapolating the p*K*_a_ values in DMSO-d_6_/H_2_O mixtures to what the p*K*_a_ would be in pure water.^25^

1 p_s_*K*_a_ + log[H_2_O]*A*/*ε* + *B*

**Fig. 3:**
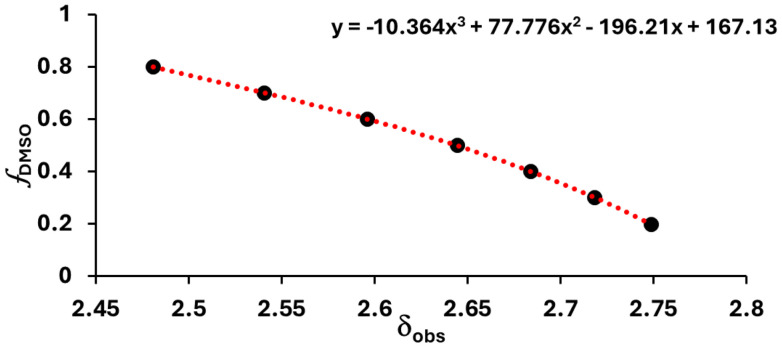
Plot of *f*_DMSO_*versus δ*_obs_ of NaMSA with respective fitting.

Where p_s_*K*_a_ is the p*K*_a_ of the compound at a specific solvent composition (SI, section S4), *ε* is the dielectric constant of the solvent and *A* and *B* are empirical constants. For that purpose, p_s_*K*_a_ of analyte, molar concentration of H_2_O, and dielectric constant of each 1D slice along the NMR tube need to be determined. H_2_O concentration was measured by determining the fraction of H_2_O present per slice and converting it to molar concentration (see SI, section S7). The dielectric constant was determined from the fractional volume of DMSO using the model of Jouyban *et al.* (see SI, section S5).^26^ The fractional volume of DMSO was determined from the ^1^H chemical shift of sodium methanesulfonate (NaMSA) which could be fitted to a third order polynomial (Fig. 3). The measurement of *f*_DMSO_ was unaffected by the presence of 5 mM HCl (SI section S6). We note that the ^1^H chemical shifts of H_2_O and DMSO are not sufficiently sensitive to solvent composition below 0.5*f*_DMSO_ due to the non-ideality of DMSO–water mixtures.^27^

The p_s_*K*_a_ of the acidic analyte is obtained by methods presented in our previous work.^28^ In brief, p_s_*K*_a_ is determined by measuring the quantity of protons transferred (*κ*) to an indicator compound with a known p_s_*K*_a_ and concentration. *κ* is related to the p_s_*K*_a_ and concentration, *C*_acid_, of the acidic analyte using eqn (2) (SI, section S1):2
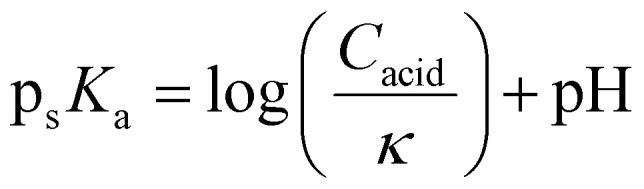


The pH of the solution throughout the different 1D spectra is determined using the indicator *via* the NMR modified Henderson–Hasselbalch equation:3
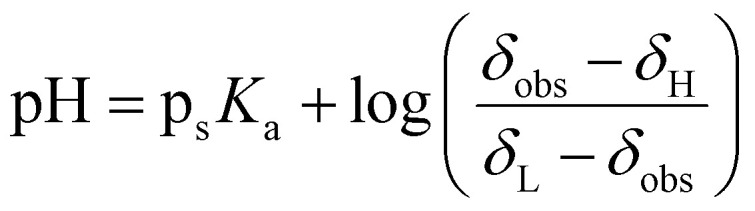
where *δ*_H_ and *δ*_L_ are the limiting chemical shifts of the indicator when it is fully protonated and fully deprotonated, respectively (SI, section S2). The limiting chemical shifts and p_s_*K*_a_ values of the indicators are experimentally determined at individual solvent compositions between 20% and 80% DMSO-d_6_ and their values obtained across the whole compositional range by polynomial interpolation (see SI, section S3 and S11). pH and *κ* can thus be determined continuously across the compositional range between 20% and 80% DMSO-d_6_ along the solvent gradient. The p_s_*K*_a_ of the analyte can be determined across the different 1D slices, and subsequently the *A* and *B* parameters can be fitted by linear regression.

**Fig. 4:**
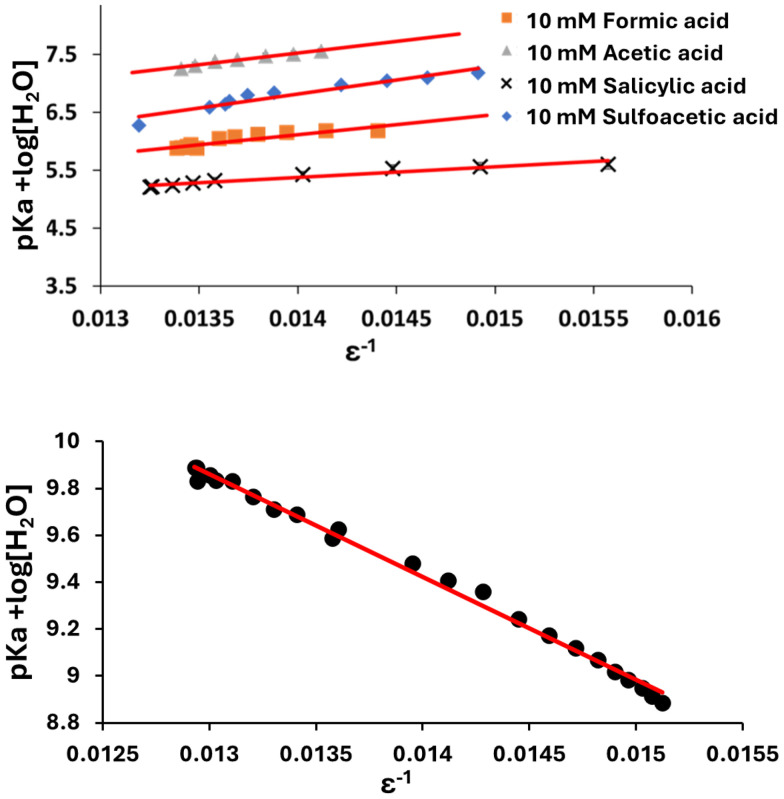
Plot of p*K*_a_ + log[H_2_O] *versus* 1/*ε* of water soluble carboxylic acids (top) and quinine hydrochloride (bottom).

The method was initially validated on water-soluble compounds (see Fig. 4). Their p*K*_a_ values in pure water were determined using the method described within 0.4 units of literature (see Table 1). As discussed in our previous work, the uncertainty in p*K*_a_, Δp_s_*K*_a_ (section S10), increases with the difference between the p*K*_a_ of the analyte.^28^ It is therefore desirable to use indicators that have p*K*_a_ values close to the expected values of the analyte. The proximity of p*K*_a_ values can be judged by comparison of inflexion points in plots of chemical shift *versus* pH.^28^

**Table 1:** Comparison of aqueous p*K*_a_ values of water soluble and sparingly soluble* compounds obtained *via* Yasuda-Shedlovsky extrapolation with literature values, with absolute difference, Δ

Analyte	Indicator	Literature p*K*_a_	Fitted p*K*_a_	Δp*K*_a_
Salicylic acid	1,2,4-Triazole	3.00 (ref. 29)	3.09	0.09
Formic acid	Sodium glycolate	3.75 (ref. 30)	3.92	0.17
Sulfoacetic acid	Sodium glycolate	4.07 (ref. 31)	4.37	0.30
Acetic acid	Sodium glycolate	4.77 (ref. 32)	5.16	0.39
Quinine hydrochloride	2-Methylimidazole	8.55 (ref. 33)	8.34	0.21
Naproxen*	Sodium formate	4.15 (ref. 34)	4.25	0.10
Indomethacin*	Sodium acetate	4.50 (ref. 35)	4.24	0.26
Furosemide*	Sodium formate, sodium glycolate	3.34 (ref. 36)	3.28	0.06

**Fig. 5:**
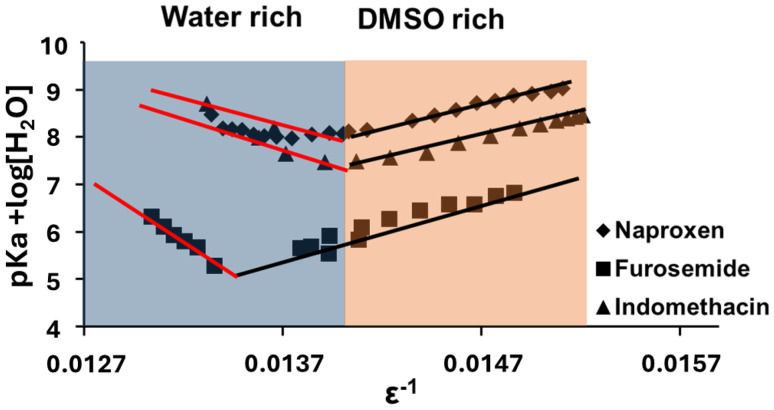
Plot of p*K*_a_ + log[H_2_O] *versus* 1/*ε* of water insoluble analytes.

We then analysed the poorly water-soluble compounds naproxen, furosemide, and indomethacin. Their behaviour was remarkably different compared to the water-soluble compounds. Fig. 5 shows how these compounds displayed a contrasting behaviour between DMSO-rich data and water-rich points. The two regimes could be represented as two lines, with better agreement with the values obtained from the literature p*K*_a_ by extrapolating from the DMSO-rich (see Table 1). Extrapolating from the water-rich data points (red lines, Fig. 5) yields aqueous p*K*_a_ values of 7.0, 9.0 and 9.3 for furosemide, indomethacin and naproxen, respectively.

**Fig. 6:**
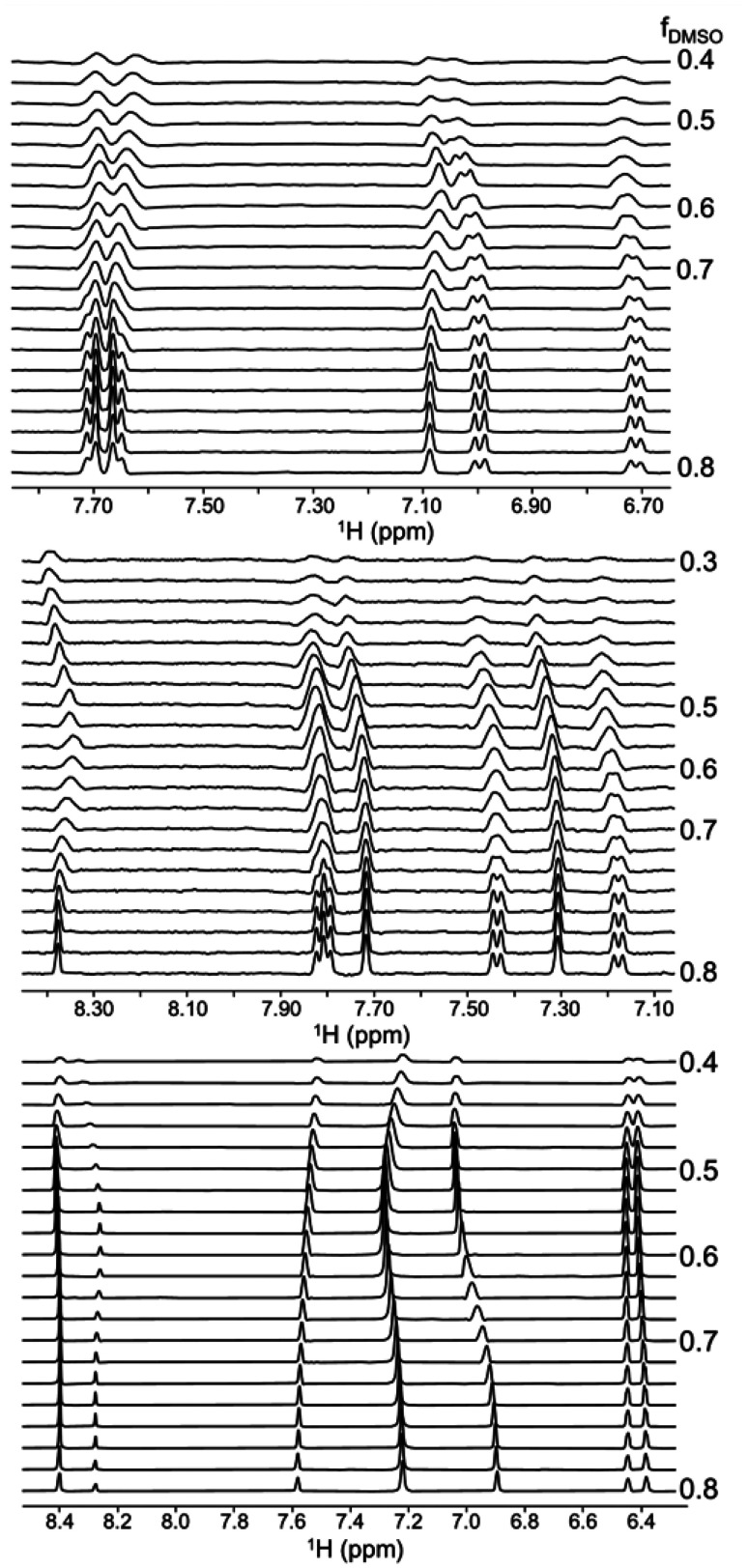
Comparison of spectra of indomethacin (top); naproxen (middle) and furosemide (bottom) along solvent gradients at the fractional volume of DMSO indicated.

These results suggest aggregation or precipitation dynamics in the water-rich regime that are not present in the DMSO-rich one. Significant broadening of the naproxen, furosemide, and indomethacin resonances was observed in the water-rich region compared to the DMSO-rich region, (see Fig. 6), suggesting that aggregation was occurring. Aggregation is a major problem in pharmaceutical assays and NMR analysis.^37^ Our approach provides a fast method to determine the minimum quantity of DMSO required to keep drug molecules in the non-associated state by monitoring the acid–base properties of the molecules. Such features are readily apparent in the Yasuda-Shedlovsky plot due to the large number of p*K*_a_ values (>10) afforded by our method from a single experiment, highlighting a key advantage of our approach over separate p*K*_a_ determinations at homogeneous solvent compositions. Solvent gradients are usable at any time during a window between 4–8 hours after preparation (Fig. S6), enabling multiple compounds to be prepared during the daytime and analysed in parallel overnight under automation on spectrometers equipped with autosamplers.

We anticipate that the method could be applied to any aqueous–organic solvent mixture. The p*K*_a_ and limiting chemical shifts of the indicators would need to be determined at three or more individual solvent ratios. The parameters of a full set of indicators (pH 2–11) could be determined in just two CSI experiments for each solvent ratio if prior literature p*K*_a_ values were available to anchor the pH scales.^20^ Calibration of an absolute reference could be performed in an additional two CSI experiments, or alternatively the parameters of the indicators could be determined using automated NMR titrations.^38^ Calibration of a suitable indicator of the solvent composition such as methanesulfonate would also be required.

### 3.2 Transferring assignment between samples in different solvents

The solvent typically affects chemical shifts, resulting in significant changes to the peak pattern of an NMR spectrum.^39^ Advanced computational methods can model them only in relatively simple cases.^40^ Because of this, resonance assignments performed in one solvent cannot be simply transferred to spectra acquired in a different solvent, necessitating the repetition of a laborious assignment procedure. In such cases, solvent-gradient experiments can be helpful.

**Fig. 7:**
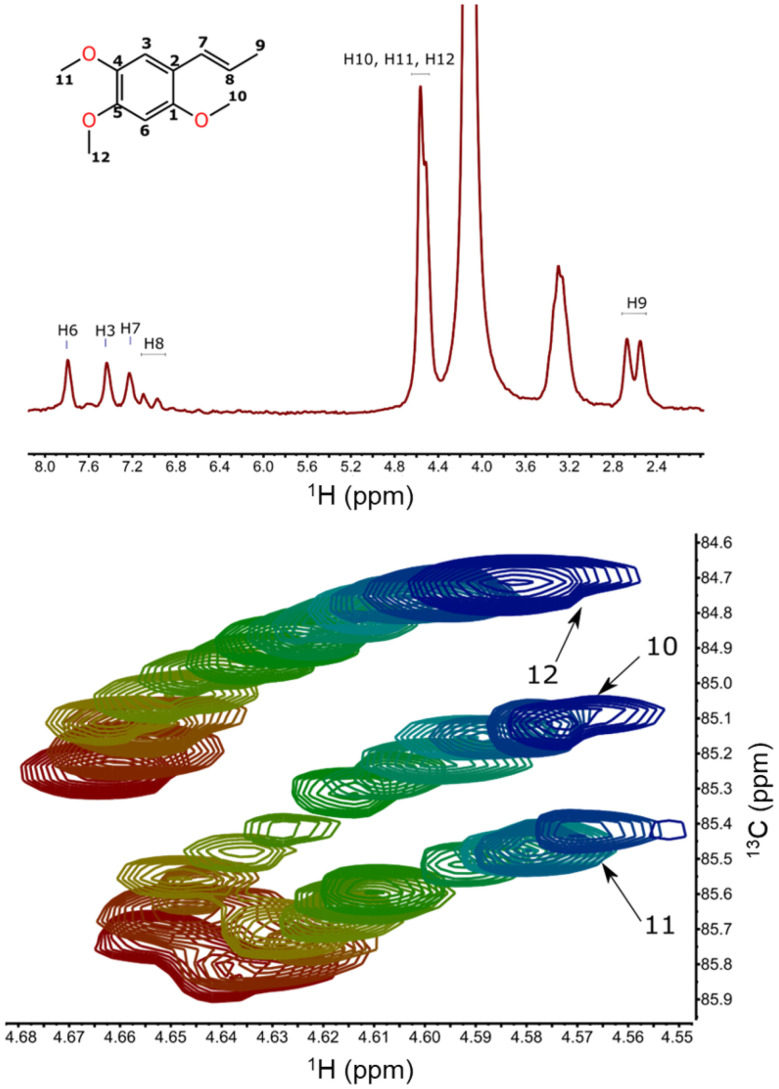
NMR spectra of α-asarone in MeOD measured on a 43 MHz benchtop NMR: ^1^H NMR (upper panel) and a stack of fifteen 2D ^1^H–^13^C HSQC spectra measured in solvent gradient DMSO : MeOD in methoxy group region. The experiments were performed 5 days after sample preparation (red corresponds to DMSO, blue – MeOD, other to mixtures).

Fig. 7 (upper panel) shows a ^1^H NMR spectrum of α-asarone acquired at 43 MHz. As can be seen, the signals from methyl groups strongly overlap, and their assignment requires time-consuming heteronuclear 2D experiments. Once the assignment is achieved in one solvent, it would be very unfavorable to repeat it in another, especially taking into account the low sensitivity of benchtop NMR. Fortunately, the solvent-gradient experiment reveals a smooth trajectory of chemical shifts, enabling the transfer of assignments. Fig. 7 (lower panel) shows superimposed fragments of fifteen 2D ^1^H–^13^C HSQC spectra acquired in a DMSO : MeOD gradient. Clearly, the peak patterns differ in pure solvents, which may make the transfer ambiguous. However, with spectra acquired in a gradient, the transfer trajectories become clear.

### 3.3 Resolving the spectrum of a mixture

The various components of a solid-state mixture, such as a drug tablet, may exhibit different solubilities in a given solvent. This effect can be used to resolve ambiguities in the spectra of mixtures and is especially useful for low-field machines.

**Fig. 8:**
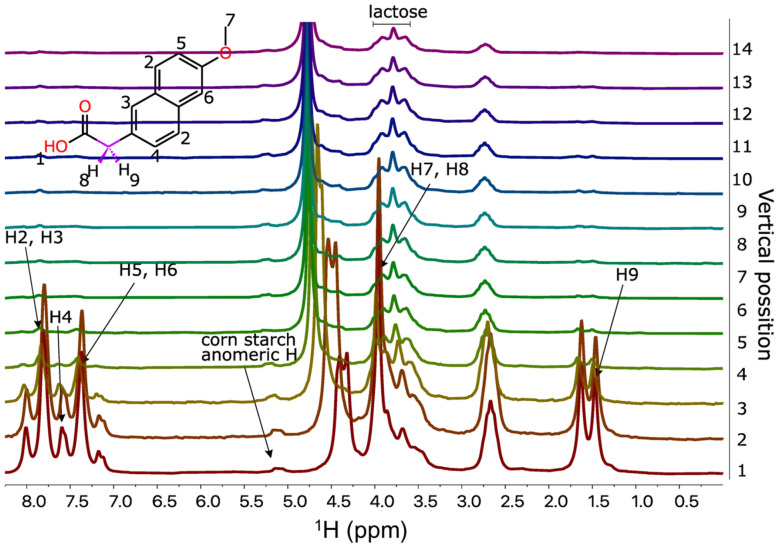
Stack of ^1^H NMR spectra of naproxen drug (200 mg naproxen in a tablet, Aflofarm Farmacja Polska Sp. z o.o.) measured on 43 MHz benchtop NMR in solvent gradient DMSO : 80% D_2_O : 20% DMSO. The experiments were performed 24 h after sample preparation (red (first one down) corresponds to DMSO, pink (last one at the top) – 80% D_2_O : 20% DMSO, other to mixtures).

Fig. 8 shows the set of spectra of naproxen drug tablet dissolved in a gradient of D_2_O and DMSO. The DMSO is an effective solvent for all main ingredients – naproxen, lactose, and starch – and thus the spectrum is very crowded. The peak overlap makes the identification of resonances impossible. However, the gradient of solvent composition creates a spectral “pseudo-dimension” that resolves peaks based on the solubility of a given component. It also allows for the identification of which peaks probably belong to the same compound, since they have to follow the same intensity change along the gradient.

## 4 Conclusions

We have demonstrated how the solvent composition across an NMR sample can be varied from DMSO to water, and DMSO to methanol *via* the diffusion of two layers of solvent. This procedure condenses tedious manual titrations and enables the measurement of key properties including p*K*_a_ values, chemical shifts and 2D correlations as a continuous function of the solvent composition in one sample. Our methods can be applied both to solutions and to semi-solid samples (naproxen tablet), promising a range of future applications from materials science to pharmaceutical analysis and organic synthesis; all areas where control and optimisation of the solvent is vital.

## Author contributions

Conceptualization: H. H., M. W., K. K., P. P.; data curation: all authors; formal analysis: H. H., M. W., K. K., P. P.; funding acquisition: M. W., K. K.; investigation, validation, and methodology: all authors; project administration and supervision: M. W., K. K.; resources: M. W., K. K.; visualization: H. H., P. P.; writing – original draft: H. H., M. W., K. K., P. P.; writing – review & editing: all authors.

## Conflicts of interest

There are no conflicts to declare.

## Supplementary Material

AN-151-D6AN00031B-s001

## Data Availability

Additional data supporting this article are available in the supplementary information (SI). Research data will be available at: https://research-portal.uea.ac.uk/en/datasets/. The pulse sequences and processing scripts are freely available on the UEA Digital Repository (https://research-portal.uea.ac.uk/en/datasets/data-for-measurement-of-the-pka-values-of-organic-molecules-in-aq/) in conjunction with our prior published work. Supplementary information: experimental and calculation procedures, plots for NMR indicators. See DOI: https://doi.org/10.1039/d6an00031b.
