# Supplementary material for: High- and low-field NMR in binary solvent gradients
Source: Analyst. 2026 Mar 20;151(9):2662–8. doi: 10.1039/d6an00031b (PMC13020772; doi:10.1039/d6an00031b)
Supplement: AN-151-D6AN00031B-s001 [file AN-151-D6AN00031B-s001.pdf]

Supplementary Information (SI) for Analyst.
This journal is © The Royal Society of Chemistry 2026

Supplementary Information for "High- and low-field NMR in binary solvent gradients"

Haider Hussain,[a], Paulina Putko,[b], Dariusz Gołowicz[c], Krzysztof Kazimierczuk,[b,†*], and Matthew Wallace,[a,*]

Contents

S1 Determination of pK_a of analyte across the NMR tube using proton transfer 1

S2 Measurement of pK_a and limiting chemical shifts of indicator compounds 2

S3 Calibrating limiting chemical shift of indicator compounds across range of solvent compositions 10

S4 Determing the pK_a of indicator compounds at any DMSO/H_2O solvent composition 10

S5 Modelling the change in dielectric constant as a function of solvent composition 10

S6 Measuring fractional volume of DMSO in presence of acid 12

S7 Determining concentration of H_2O across the NMR tube 13

S8 Solvent gradient optimum time determination 13

S9 Using multiple indicators to determine pH and analyte p_sK_a 14

S10 Estimation of uncertainty in the determination of aqueous pK_a 15

S11 Fitting plots to determine pK_a , δ_H and δ_L of indicator compounds at any DMSO/H_2O solvent composition. 16

S12 Creation of solvent composition gradients between DMSO and $CHCl_3$ and analysis by high-field NMR 22

References 22

S1 Determination of pK_a of analyte across the NMR tube using proton transfer

Determination of pK_a is performed via measuring the quantity of protons transferred from the analyte to an indicator.[1] The original method consisted in measuring the quantity of acidic protons removed from the analyte or "protons transferred" (symbolised as κ) across the NMR tube (at different concentrations of basic indicator and pH) using equation S1 and then obtaining the pK_a along with the concentration of acid via linear regression of the data to equation S2:

$$\kappa = C_{indicator}\frac{\delta_{obs}-\delta_L}{\delta_H-\delta_L} \tag{S1}$$

$$\kappa = \frac{C_{acid}}{10^{p_sK_a-pH}+1} \tag{S2}$$

Where p_sK_a is the pKa of the compound at a specific solvent composition, $_obs$, $_H$ and $_L$ are the experimentally observed and limiting chemical shifts of the indicator when it is fully protonated (L) and fully deprotonated (H), respectively. C_{acid} and $C_{indicator}$ are the concentrations of acidic analyte and basic indicator, respectively. Given that the pK_a of the analyte

[0a] *School of Chemistry, Pharmacy and Pharmacology, University of East Anglia, Norwich Research Park, Norwich, NR4 7TJ, UK*
[0b] *Centre of New Technologies, University of Warsaw, Banacha 2C, Warsaw, 02-097, Poland.*
[0c] *Institute of Physical Chemistry, Polish Academy of Sciences, Kasprzaka 44/52, Warsaw, 01-224, Poland.*
[0†] *email:k.kazimierczuk@cent.uw.edu.pl*
[0*] *email:Matthew.Wallace@uea.ac.uk*

changes across the NMR tube due to changes in the solvent composition, it is not possible to perform a linear regression as previously described. For this setup, the pK_a of the analyte can be calculated directly by calculating κ for each 1D slice (solvent composition) by measuring the indicator chemical shift and solving for pK_a using equation S1 and S2. Assuming that the quantity of protons transferred to the indicator are equal to the quantity of protons transferred from the analyte, equation S2 can be rearranged to equation S3 (equation 2 in the main paper):

$$p_sK_a = log(\frac{C_{acid}}{\kappa} - 1) + pH \tag{S3}$$

In our experiments, the concentrations of analyte and indicator are made equal for convenience, as this makes $C_{indicator}$ of equation S1 equal to C_{acid} of equation S2 which effectively cancels out the concentration terms from the equations S1 and S2. p_sK_a of the analyte for each slice is obtained *via* determination of pH using the NMR modified Henderson-Hasselbalch equation and the proton transfer through equation S1, obtaining p_sK_a from equation S3.

S2 Measurement of pK_a and limiting chemical shifts of indicator compounds

Measurement of pK_a and limiting chemical shift of indicator compounds across multiple DMSO/H_2O solvent compositions was performed using the method of Wallace et al.[2] The first step is to determine the absolute pK_a values of two acids which undergo autodissociation to a significant when dissolved in the solvent mixture of interest: 2,6-dihydroxybenzoic acid (80/20 DMSO/H_2O) and maleic acid (20/80 DMSO/H_2O). Indicator data at 50/50 DMSO/H_2O was taken directly from Wallace et al.[2], with the exception of formate which was determined in the present work (Figure S1 below).

Determination of absolute p_sK_a values of 2,6-dihydroxybenzoic acid and maleic acid

The pK_a of maleic acid in 20/80 DMSO/H_2O and 2,6-dihydroxybenzoic acid (2,6-DHB) in 80/20 DMSO/H_2O were determined by creating concentration gradients of these acids within the solvent mixture in the absence of bases and measuring their ^{1}H chemical shift relative to DSS as a function of their concentration. For 20/80 DMSO/H_2O, 6.5 mg of maleic acid was transferred into a Wilmad-528-PP NMR tube using a 9 inch tip Pasteur pipette and four 2 mm diameter glass beads placed on top. A solution of DSS (10 mM) in 20/80 DMSO/H_2O was then layered on top of the beads to a height of 40 mm from the absolute tube base and the sample left for 8 hours for the concentration gradient to develop. For 80/20 DMSO/H_2O, 8.9 mg of 2,6-dihydroxybenzoic acid was transferred to an NMR tube and four 2 mm diameter glass beads placed on top. A solution of DSS (10 mM) in 80/20 DMSO/H_2O was then placed on top and the sample left to stand for 33 hours before analysis. The NMR experiments were performed as described for 50/50 DMSO/H_2O in Wallace et al.[2] In the absence of other acids or bases, the fraction of 2,6-DHB or maleic acid in its deprotonated state, f_L, is given by eq S4:

$$f_L = \frac{-K_a + \sqrt{K_a^2 + K_aC}}{2C} \tag{S4}$$

Where K_a is concentration-based and C is the total concentration of 2,6-DHB or maleic acid at each position along the sample. Assuming a fast exchange on the ^{1}H NMR time scale, the observed chemical shift of the acid, δ_{obs}, is given by:

$$\delta_{obs} = f_L\ \delta_L + \ (1 - f_L)\delta_H \tag{S5}$$

Where δ_H and δ_L are the limiting chemical shifts of the protonated and deprotonated states, respectively. C is obtained by integrating the ^{1}H resonance of the 3,5-position of the aromatic ring of 2,6-DHB against the methyl resonance of DSS at each position along the sample using CSI. The molar ionic strength, I, of the solution at each position is calculated as the sum of the concentrations of DSS and dissociated 2,6-DHB. δ_H and δ_L and K_a are then fitted as free variables with respect to equation S5. K_a is converted to thermodynamic K_a using activity coefficient γ. The activity coefficient of a univalent ion, is obtained from eq S6:

$$log(\gamma) = \frac{-A\sqrt{I}}{1 + B\sqrt{I}} \tag{S6}$$

Where A and B are parameters to modulate what the activity coefficient will be depending on the medium. A and B can be calculated:[3]

$$A = 1.826 \times 10^6\ (\varepsilon T)^{\ (-3/2)} \tag{S7}$$

$$B = r \times 50.3 \times (\varepsilon T)^{(-1/2)} \tag{S8}$$

Where ε is the dielectric constant, r is radius of the ion (Å), which is assumed to be 4 Å, and T is the absolute temperature. Thermodynamic Ka is then obtained *via* the following equation:

$$K_a = K_a^{\text{app}} \frac{\gamma_{\text{H}^+} \gamma_{\text{A}^-}}{\gamma_{\text{HA}}} \tag{S9}$$

Where γ_{H^+} is activity coefficient of the hydrogen ion, γ_{A^-} is activity coefficient of the conjugate base and γ_{HA} activity coefficient of the undissociated acid.

Determination of absolute p_sK_a values of 1,2,4-triazole

The concentration gradients employed to determine the absolute p_sK_a values of 2,6-dihydroxybenzoic acid and maleic acid were then repeated in the presence of 1,2,4-triazole (40 mM) and the fitted pK_a values of maleic acid and 2,6-dihydrozybenzoic acid used to determine the pK_a values of 1,2,4-triazole as described in Wallace et al.[2] For 20/80 DMSO/H_2O, 6.0 mg of maleic acid was weighed into the NMR tube and the sample left for 9 hours for the concentration gradient to develop. For 80/20 DMSO/H_2O, 8.3 mg of 2,6-dihydroxybenzoic acid was weighed out and the sample stood 33 hours for the gradient to develop.

Determination of p_sK_a values of of indicators to form a ladder

Having determined the limiting chemical shifts and p_sK_a of 1,2,4-triazole, indicator ladders were constructed where 2D ^{1}H CSI NMR experiments were run with multiple indicators to determine the p_sK_a, δ_H and δ_L of each indicator to enable the determination of pH across wide ranges, as described in our previous work[2]. The uncertainty in the fitted pK_a was calculated using a modified procedure to that described in Section S7.2 of that work. In 20/80 DMSO/H_2O, the parameters of dimethylglycine, glycolate and acetate were determined by layering 40 uL of 0.5 M ammonia on top of 500 uL of a solution containing sodium acetate (10 mM), sodium glycolate (10 mM), 1,2,4-triazole (10 mM), 2-methylimidazole (10 mM), HCl (60 mM), sodium salicylate (2 mM), dimethylglycinate (2 mM), furosemide (2 mM) and DSS (0.4 mM). The sample was stood for 33 hours before analysis for a pH gradient to develop. To determine the parameters of imidazole, 2-methylimidazole and 4-cyanophenol, 40 uL of 0.5 M ammonia was layered on top of 500 uL of a solution containing sodium acetate (10 mM), sodium glycolate (10 mM), 4-cyanophenolate (2 mM), imidazole (10 mM), 2-methylimidazole (10 mM), HCl (60 mM), dimethylglycinate (2 mM), bromothymol blue (0.2 mM) and DSS (0.4 mM). The sample was left to stand for 37 hours prior to analysis. δ_L of each indicator was taken as the value measured in a solution of the basic form in the absence of acid. The pH was determined from the known parameters of the indicators using Equation S10, and δ_H and pK_a determined by fitting the chemical shift of the new indicator to Equation S11.

In 80/20 DMSO/H_2O, the parameters of 3,5-dimethylpyrazole, 2,6-lutidine, imidazole, glycolate and acetate were determined by layering a solution containing 10 mM sodium glycolate, acetate, 20 mM 3,5-dimethylpyrazole, 2,6-lutidine, 1,2,4-triazole, imidazole, 2 mM dimethylglycinate, salicylate and 0.4 mM DSS was layered on top of 4.3 mg 2,6-DHB and the sample stood for 62 hours prior to analysis. To determine the parameters of dimethylglycinate (upper pK_a), a solution containing 20 mM sodium glycolate, acetate, benzylamine, 10 mM 4-cyanophenolate, dimethylglycinate, 2 mM methylammonium chloride, NaOH and 0.4 mM DSS was layered on top of 5 mg of 2,6-DHB and the sample stood for 112 hours prior to analysis. To determine the parameters of 2-methylimidazole and formate in this solvent mixture, a solution containing 20 mM sodium glycolate, acetate, formate, 3,5-dimethylpyrazole, 2-methylimidazole and 0.4 mM DSS was layered on top of 4.2 mg of 2,6-DHB and the sample stood for 18 hours before analysis. In the CSI experiments, 6 ms triangular shaped pulses were used to selectively invert the water magnetisation.

The uncertainty in a new indicator was taken as the square root of the sum of the squares of the largest uncertainty in pK_a of the known indicators plus the average difference between the pH calculated using Equation 12 of our previous work[2] with only the known indicators, and pHi of the new indicator, for all rows where pHi could be calculated.

Determination of p_sK_a of formic acid

For determination of limiting chemical shifts and pK_a of formate in 80/20 and 50/50 and H_2O/DMSO a different approach was used. Instead proton NMR CSI experiments were run with analyte solution containing 10 mM of sodium formate, 0.2 mM of DSS and 20 mM of sodium methanesulfonate. A solution of acetic acid (2 M, 30 μL) was then pipetted at the bottom of the NMR tube and four glass beads were placed in the solution. An aliquot of the analyte solution was then drawn up in a 9 in. Pasteur pipet and gently layered on top to a height of 40-50 mm from the base of the NMR tube. This approach allows for an acid concentration gradient to be produced in the NMR tube causing for a variation in pH across the NMR tube. The pH is then measured across the NMR tube using equation S10 with acetic acid as the pH indicator:

$$pH = p_sK_a + log(\frac{\delta_{obs} - \delta_H}{\delta_L - \delta_{obs}}) \tag{S10}$$

rearranging equation S10 to make δ_{obs} the subject of the equation:

$$\delta_{\text{obs}} = \frac{\delta_{\text{H}}\, 10^{(pK_{\text{a}} - \text{pH})} + \delta_{\text{L}}}{1 + 10^{(pK_{\text{a}} - \text{pH})}} \tag{S11}$$

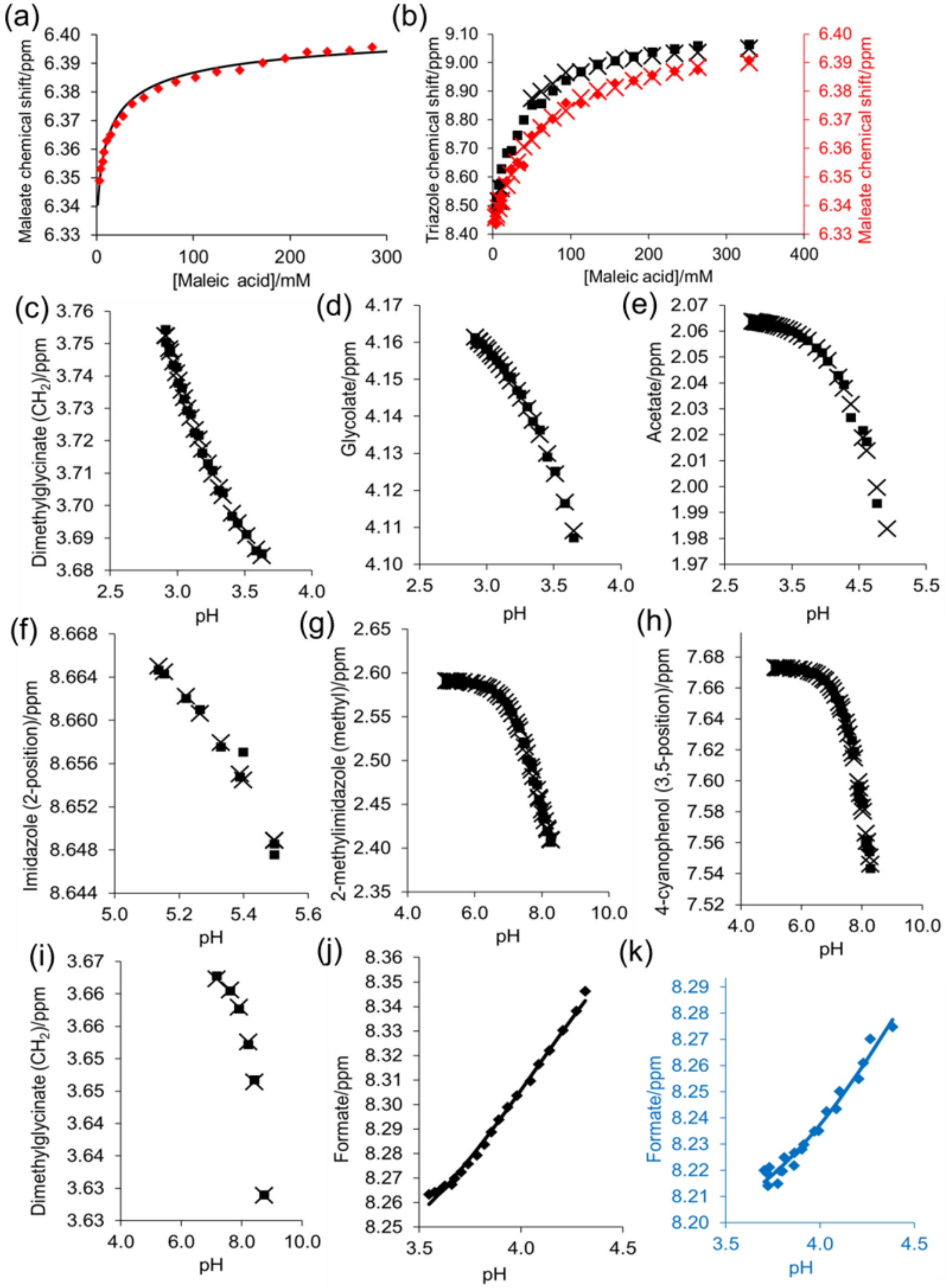

Figure S1: Plots used to determine parameters of NMR indicators in 20/80 DMSO/H_2O: (a) Plot of ^{1}H chemical shift of maleic acid versus concentration. Fits to S4 and S5 (solid line). (b) Plots of ^{1}H chemical shifts of maleate and 1,2,4-triazole as a function of the concentration of maleic acid. Fit to Equations 2, 7 and 8 of Wallace et al.[2] (cross). Calibration plots of observed chemical shifts of indicators (solid symbols) versus pH, fits to Equation S11 (cross), formate (solid line): dimethylglycine (c), glycolate (d), acetate (e), imidazole (f), 2-methylimidazole (g), 4-cyanophenol (h), dimethlyglycine (deprotonation step, i) and formate (j). (k) Calibration plot for formate in 50/50 DMSO/H_2O (blue).

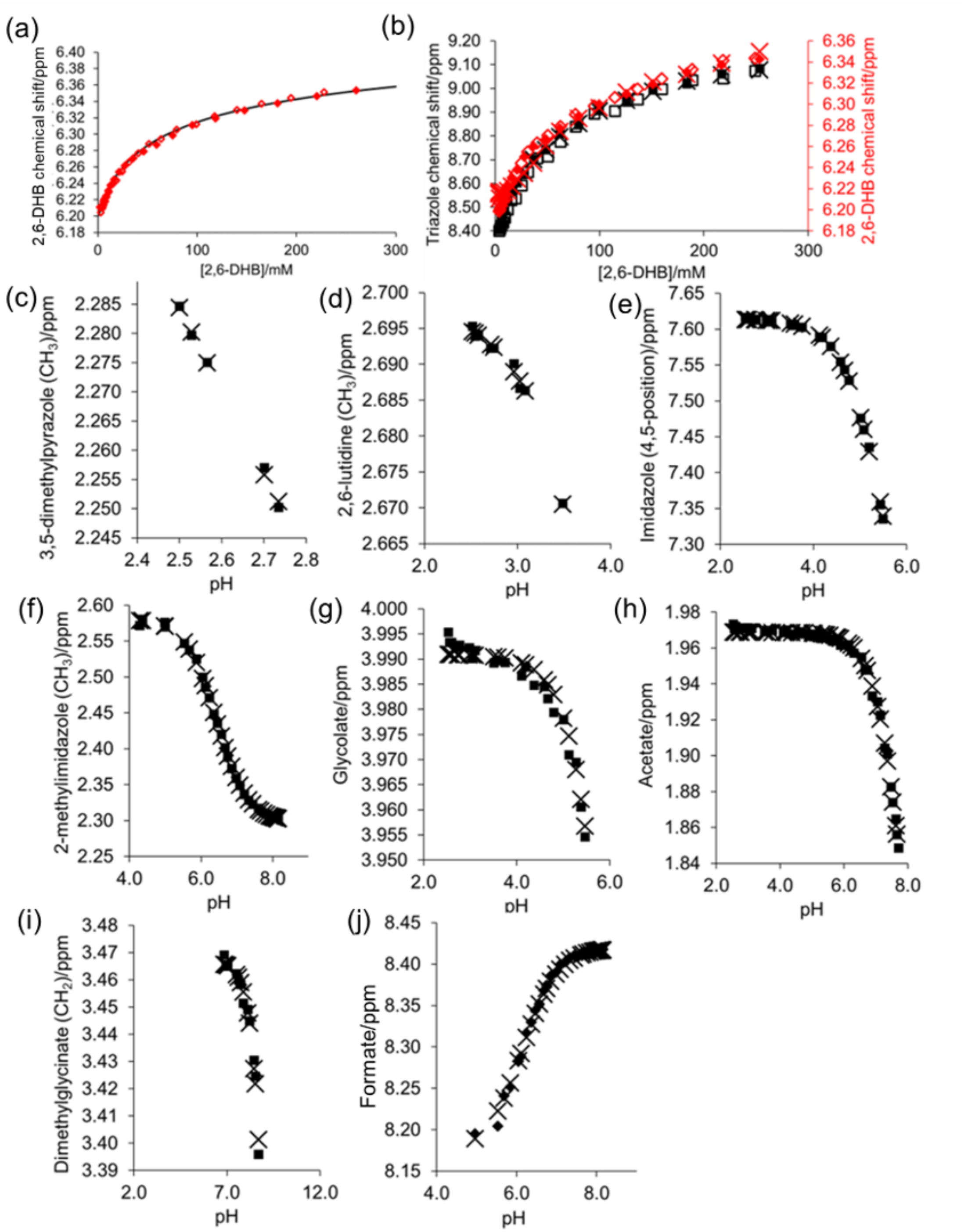

Figure S2: Plots used to determine parameters of NMR indicators in 80/20 DMSO/H_2O: (a) Plot of ^{1}H chemical shift of 2,6-DHB versus concentration determined by integration against DSS (solid symbols) or dimethylsulfone (20 mM, open symbols). Fits to S4 and S5 (solid line). (b) Pots of ^{1}H chemical shifts of 2,6-DHB and 1,2,4-triazole as a function of the concentration of 2,6-DHB . Fit to Equations 2, 7 and 8 of Wallace et al.[2] (cross). Calibration plots of observed chemical shifts of indicators (solid symbols) versus pH, fits to Equation S11 (cross): 3,5-dimethylpyrazole (c), 2,6-lutidine (d), imidazole (e), 2-methylimidazole (f), glycolate (g), acetate (h), dimethylglycinate (i) and formate (j).

δ_H and pK_a of formate are then fitted as free parameters for equation S11 with δ_{obs} and pH measured experimentally across the NMR tube. δ_L was determined in solutions containing 10 mM sodium formate and 0.2 mM DSS and fixed during the data fitting. This was done for 80/20 and 50/50 H_2O/DMSO solvent composition. The pK_a δ_L , δ_H for formate in 50/50 H_2O/DMSO were obtained as 8.3867, 8.1851 and 4.45±0.31, respectively, comparing to the pK_a value of 4.73 reported by Mollin et al.[4] The uncertainty in the pK_a of formate is assumed equal to the uncertainty in the pK_a of acetic acid. The parameters of formate in 20/80 H_2O/DMSO were determined in the sample used to determine the parameters of 2-methylimidazole in this solvent mixture.

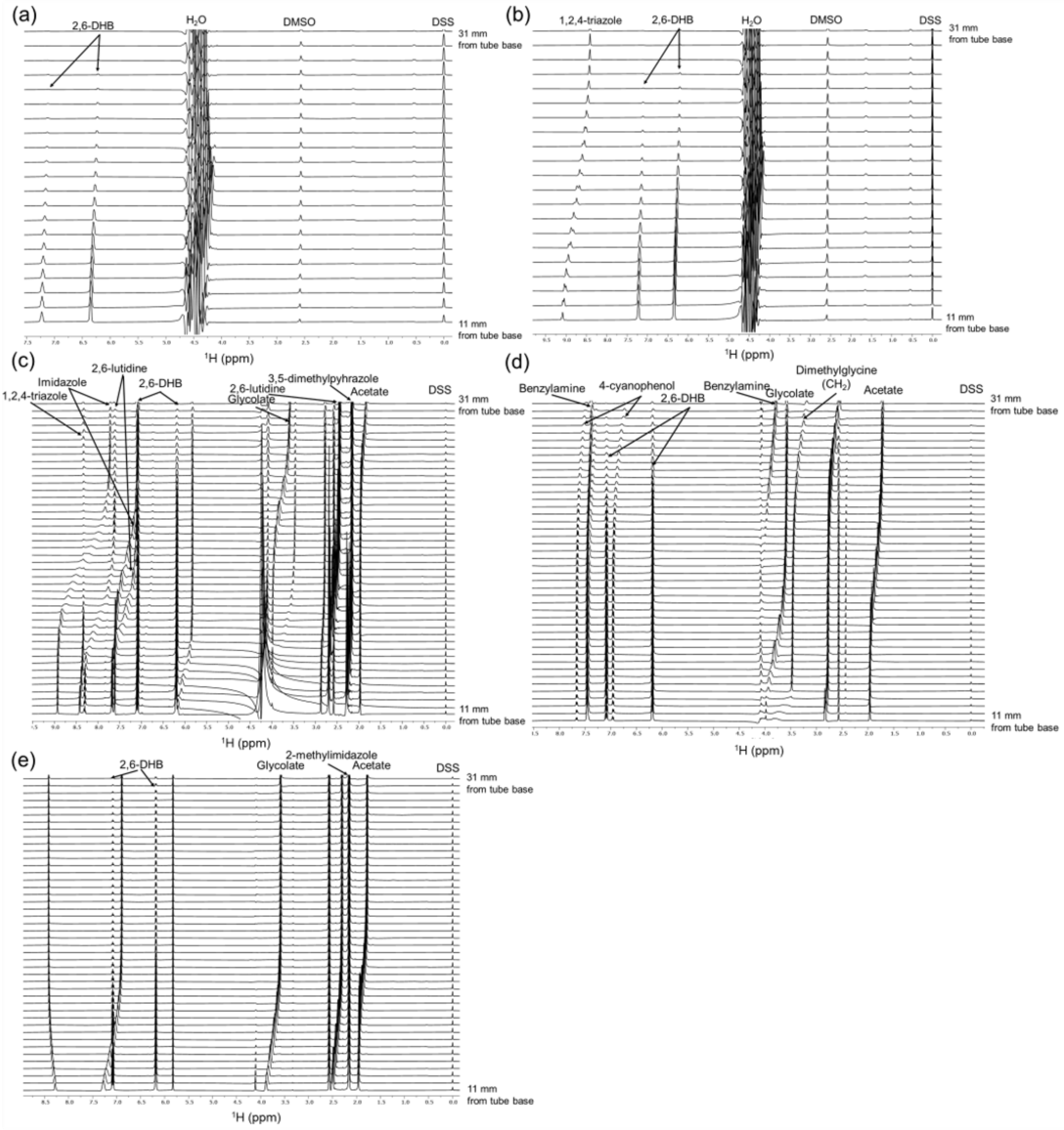

Figure S3: NMR spectra used to determine parameters of NMR indicators in 80/20 DMSO/H_2O: (a) 2,6-DHB acid. (b) 2,6-DHB and 1,2,4-triazole. (c) 3,5-dimethylpyrazole, 2,6-lutidine, imidazole, glycolate and acetate. (d) dimethylglycinate. (e) 2-methylimidazole and formate.

Extracted δ_L , δ_H and pK_a values of all indicators are presented in Tables S1 and S2.

Indicator	δ_L	δ_H	pKa	Literature pKa
2,6-DHB	6.1957	6.4735	1.35±0.02	-
1,2,4-triazole	8.3271	9.3484	1.42±0.13	-
3,5-dimethylpyrazole (methyl)	2.1557	2.4015	2.45±0.13	-
2,6-lutidine (methyl)	2.4377	2.6977	4.32±0.13	-
Sodium glycolate	3.5680	3.9910	6.61±0.16	6.94[5]
Sodium formate	8.4196	8.1738	6.27±0.17	6.42[6], 6.51[4]
Imidazole	7.0800	7.6140	5.37±0.13	-
2-methylimidazole	2.3179	2.5789	6.34±0.17	-
Sodium acetate	1.7127	1.9688	7.86±0.17	7.27[7], 8.00[8]
Dimethylglycinate	2.7400	3.4668	9.78±0.18	-

Table S1: Parameters of indicators at 20/80 H_2O/DMSO

Indicator	δ_L	δ_H	pKa	Literature pKa
Maleic acid	6.3334	6.4074	2.10±0.06	-
1,2,4-triazole	8.3590	9.1079	2.42±0.27	-
Dimethylglycinate (CH2)	2.8851	2.9312	2.55±0.27	-
Sodium glycolate	3.8702	4.1765	4.28±0.27	-
Sodium formate	8.4170	8.2127	4.08±0.28	4.13[4]
Imidazole	7.7564	8.6781	6.87±0.28	-
2-methylimidazole	2.3359	2.5915	7.76±0.28	-
4-cyanophenolate	7.4367	7.6734	8.32±0.28	-
Sodium acetate	1.8674	2.0651	5.16±0.28	4.90[9]

Table S2: Parameters of indicators at 80/20 H_2O/DMSO

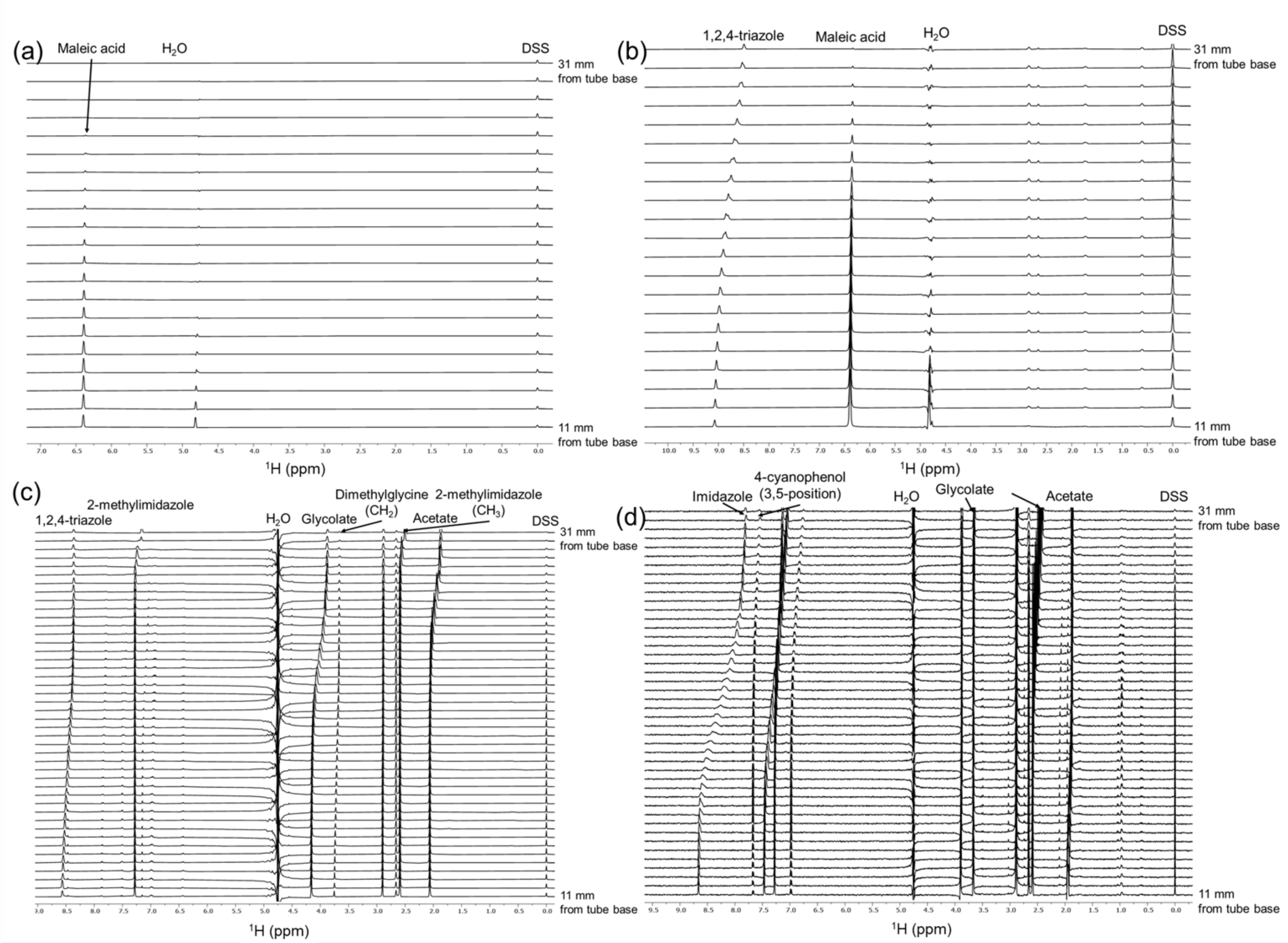

Figure S4: NMR spectra used to determine parameters of NMR indicators in 20/80 DMSO/H_2O: (a) 2,6-DHB acid. (b) 2,6-DHB and 1,2,4-triazole. (c) 3,5-dimethylpyrazole, 2,6-lutidine, imidazole, glycolate and acetate. (d) dimethylglycinate. (e) 2-methylimidazole and formate.

S3 Calibrating limiting chemical shift of indicator compounds across range of solvent compositions

For purposes of this experimental setup, the limiting chemical shifts of the indicator need to be known for any given DMSO/H_2O solvent composition. This requires an equation that models the limiting chemical shift of an indicator as a function of the solvent composition. In order to obtain this equation, the limiting chemical shifts of indicators were determined for 0/100, 80/20, 50/50 and 20/80 DMSO/H_2O fractional volume using the method described in Section S2. The values obtained are then fitted to a polynomial equation with respect to mole fraction of DMSO (See Section S11 for fitting plots), by measuring the fractional volume of DMSO using the method described previously. The limiting chemical shifts of the indicator are determined for each 1D spectrum using the specific fractional volume of DMSO.

S4 Determing the pK_a of indicator compounds at any DMSO/H_2O solvent composition

The pK_a of indicator compounds needs to be known for any given DMSO/H_2O solvent composition. Thus, a relation for the pK_a of the indicator compound as a function of the fractional volume of DMSO is needed. Such a relation is obtained by utilizing the Yasuda-Shedlovsky equation for the pK_a values of the indicator already obtained for 80, 50, and 20 % fractional volume of DMSO using the method described in section S2. By fitting the Yasuda-Shedlovsky equation for the pK_a values already determined the slope and y-intercept of the linear equation is obtained (A and B parameters, respectively). This allows for determination of pK_a of indicator as a function of the dielectric constant which can be determined via measuring the fractional volume of DMSO as described previously across the different 1D spectra by determining the dielectric constant specific for that spectra and inputting into the Yasuda-Shedlovsky equation (see Section S11 for fitting plots).

S5 Modelling the change in dielectric constant as a function of solvent composition

Modelling the change in dielectric constant as a function of solvent composition was done using the model utilized by Jouyban et al, which models the dielectric constant of a binary solvent mixture:[10]

$$ln\ (\varepsilon)\ =\ \varphi_1\ ln(\varepsilon_1)\ +\ \varphi_2\ ln(\ \varepsilon_2) +\ \varphi_1\ \varphi_2 \sum_{i=0}^{2} K_i\ (\varphi_1 - \varphi_2)^i \tag{S12}$$

Where ε_1 is dielectric constant of H_2O, ε_2 is dielectric constant of DMSO, φ_1 is mole fraction of H_2O , φ_2 is mole fraction of DMSO and K_i are optimisation constants. The model was calibrated for DMSO/H_2O solutions by fitting the data of Płowaś et al.[11] to the equation of Jouyban et al.[10] to obtain the solvent-dependent optimisation constants (see table S4). Mole fractions are then converted to volume fractions using equation S13:[11]

$$f_{DMSO} = \frac{\chi_{DMSO} \cdot 100 \cdot \frac{\rho_{\mathrm{H_2O}}}{Mr_{\mathrm{H_2O}}}}{\frac{\rho_{\mathrm{DMSO}}}{Mr_{\mathrm{DMSO}}} \cdot (1 - \chi_{DMSO}) + \chi_{DMSO} \cdot \frac{\rho_{\mathrm{H_2O}}}{Mr_{\mathrm{H_2O}}}} \tag{S13}$$

Where χ_{DMSO} is mole fraction of DMSO, $\rho_{\mathrm{H_2O}}$ is density of H_2O and ρ_{DMSO} is density of DMSO (1.0 and 1.1 g/mL respectively). Dielectric constant values obtained using the equation were compared with experimental values with reasonable accuracy obtained (see figure S5 and table S3).

χ_{DMSO}	f_{DMSO}	Literature ε	Fitted ε
0.1536	0.417	75.97	75.96
0.2000	0.496	74.83	74.75
0.2496	0.567	73.52	73.41
0.3000	0.628	71.86	71.96
0.3505	0.680	70.29	70.42
0.4000	0.724	68.09	68.80
0.4975	0.796	65.13	65.34
0.5	0.798	67.93	65.25

Table S3: Fitted values of ε with respect to mole or volume fraction of DMSO in a DMSO-H_2O solution

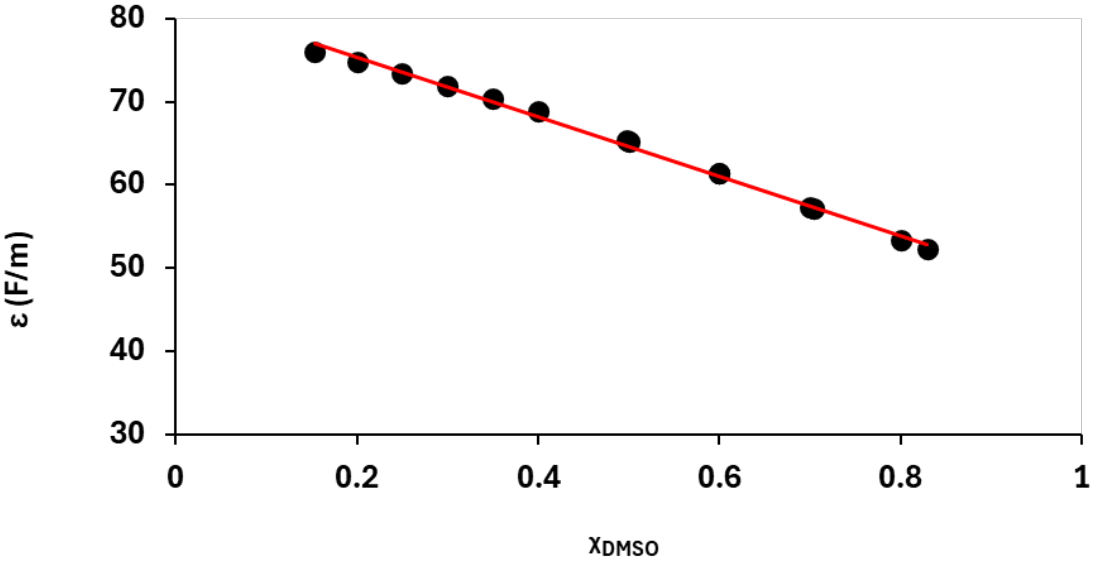

Figure S5: Plot of ε as a function of the mole fraction of DMSO with its respective fitting to the data of Płowaś et al.[11]

Optimsation constant	Fitted value
K_0	0.263
K_1	-0.066
K_2	-0.146

Table S4: Fitted optimsation constants values for equation S12

S6 Measuring fractional volume of DMSO in presence of acid

The fractional volume of DMSO is measured using sodium methanesulfonate as solvent indicator (Figure 3, main text). Sodium methanesulfonate was used due to its low pK_a value (-2.6) thus for the pH range of interest for our experiments the chemical shift of sodium methanesulfonate is independent of pH.[12] Thus, by measuring the chemical shift of sodium methanesulfonate and seeing the change in chemical shift as a function of fractional volume of DMSO an equation that relates the chemical shift of sodium methanesulfonate to fractional volume of DMSO is obtained. For that end a series of 1D ^{1}H NMR experiments are performed with 20 mM sodium methanesulfonate, 0.2 mM DSS and each containing different DMSO/H_2O solvent composition ranging from 80% fractional volume of DMSO to 20% fractional volume of DMSO.

Polynomial fitting is done with respect to this data with the chemical shift of sodium methanesulfonate as the independent variable and the fractional volume of DMSO as the dependent variable and a polynomial fit is obtained. The plot and fitting equation are provided on Figure 3. In order to test the polynomial a 1D ^{1}H NMR experiment was run with 20 mM sodium methanesulfonate, 0.2 mM DSS with a fractional volume of DMSO of 0.45 and the polynomial predicted the value with reasonable accuracy (see Table S5). Furthermore to check for whether the indicator is insensitive to pH changed we repeated the experiment in the presence of 5 mM HCl to and used the polynomial to predict the solvent composition and reasonable accuracy was obtained.

[HCl]/mM	f_{DMSO}	Measured f_{DMSO}
5	0.45	0.443
0	0.45	0.446

Table S5: f_{DMSO} measurement with NaMSA in presence and absence of HCl

S7 Determining concentration of H_2O across the NMR tube

The concentration of H_2O across the NMR tube was obtained by dividing the fractional volume of H_2O (f_{H_2O}) for each slice by the molecular weight of water (MW_{H_2O}) (equation S14). The partial molar volume of H_2O is assumed equal to 18 ml/mol across the composition range. Although the partial molar volume is sightly lower at 20% H_2O (16.6) [13], assumption of a constant molar volume has negligible impact (0.035 log units) on log[H_2O] in the Yasuda-Shedlovsky equation.

$$M_{H_2O} = \frac{f_{H_2O}}{\bar{V}_{H_2O}} \quad \text{(S14)}$$

S8 Solvent gradient optimum time determination

^{1}H CSI NMR experiments were run with 320 ul of 80/20 DMSO H_2O fractional volume solution at the bottom of the NMR tube with 320 ul of 20/80 DMSO H_2O fractional volume solution on top. Both solutions contain 0.2 mM of DSS, 20 mM of sodium methanesulfonate. Data was recorded every twenty minutes for an eight hour period and another experiment was run 24 hours after preparation. Results show that within 3 hours 40 minutes to 8 hours from sample preparation the solvent gradient has a good compromise between it having a smooth linear change of fractional volume with respect to chemical shift of sodium methanesulfonate whilst also having the sufficient variation to induce significant changes to analyte pK_a (see figure S6).

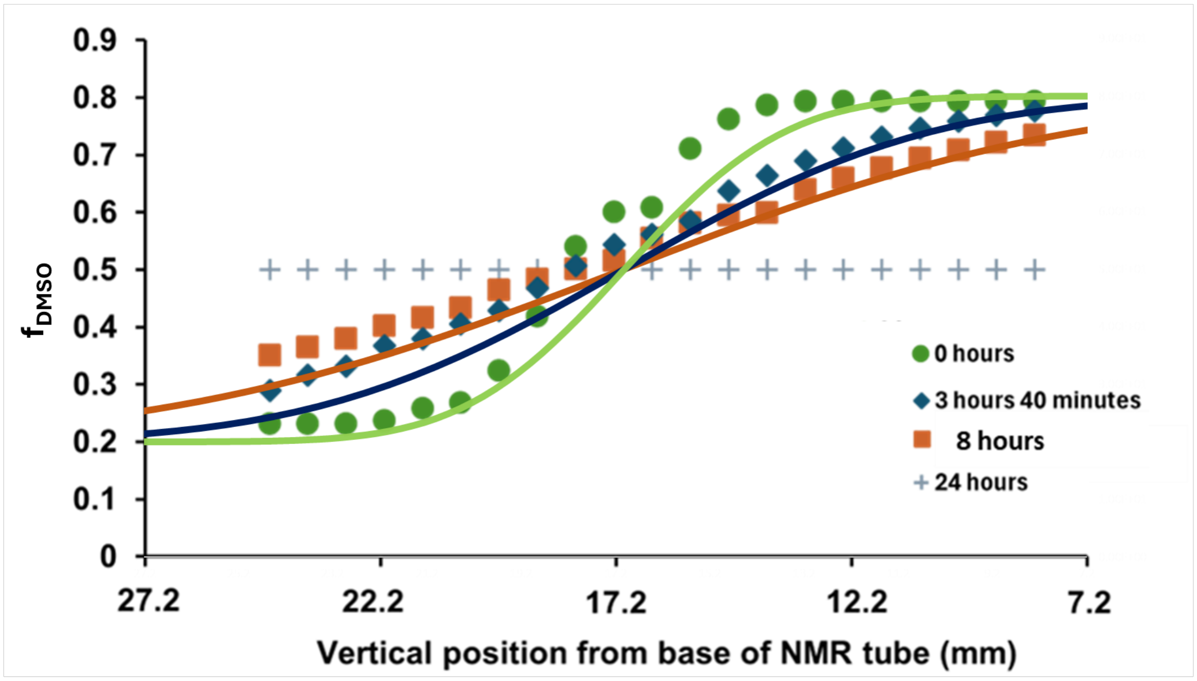

Figure S6: Plot of f_{DMSO} versus height from base of NMR tube at different times since preparation of the sample. Solid lines are predictions using Equation S15: green (1 hour), blue (3 hours 40 minutes), brown (8 hours).

The solvent composition along the gradient may be predicted using equation S15.[14]

$$f_{DMSO} = 0.5(f_{top}erfc(\frac{-z}{2\sqrt{Dt}}) + f_{bottom}erfc(\frac{z}{2\sqrt{Dt}})) \tag{S15}$$

where f_{top} and f_{bottom} are the inital volume fractions of DMSO in the two layers that are layed on top of each other. D is the diffusion coefficient of the solvent (assume 0.001 mm^2s^{-1}).[15 16]. z is the vertical distance above the boundary between the two solutions. This equation gives reasonable agreement with the observed evolution of the gradient.

S9 Using multiple indicators to determine pH and analyte p_sK_a

Using one indicator can be impractical as it could be difficult to match the pK_a of an indicator to the analyte. To increase the practicality of this technique, more than one indicator can be used simultaneously (furosemide) as large differences between the indicator and analyte's pK_a can lead to larger uncertainty in measurement. Therefore, using multiple indicators with varying values of pK_a provides an averaged pH measurement that minimizes the uncertainty generated by any individual indicator. The experimental setup is such that the sum of the protons transferred to the indicators is equal to the proton transferred by the analyte. Using equations S1 and S2 which are the equations for measuring the pK_a of one indicator, we can sum these to derive equation S16:

$$\frac{C_{\text{analyte}}}{1+10^{\text{pKa}-\text{pH}}} = \sum_{i=1}^{n} C_i \times \frac{\delta_{\text{obs},i} - \delta_{L,i}}{\delta_{H,i} - \delta_{L,i}} \tag{S16}$$

where n is the number of indicators. Given that the indicator's uncertainty with respect to pH measurement increases as pH is further from the indicator's pK_a, it is necessary to use a sensitivity-weighted average pH value when calculating κ and p_sK_a. This is done by calculating the sensitivity S_i of each indicator with respect to pH through equation S17:

$$S_i = \frac{(\delta_L - \delta_{\text{obs}})(\delta_{\text{obs}} - \delta_H)}{\delta_H - \delta_L} \tag{S17}$$

The pH value at each position along the sample is calculated as the sensitivity-weighted average of pH:

$$\text{pH} = \frac{\sum_{i=1}^{n} S_i \, \text{pH}_i}{\sum_{i=1}^{n} S_i} \tag{S18}$$

S10 Estimation of uncertainty in the determination of aqueous pK_a

The uncertainty in analyte p_sK_a across different slices is obtained by propagation of uncertainty analysis:

$$\Delta p_sK_a = \sqrt{\left(\frac{\partial p_sK_a}{\partial pH}\right)^2 \Delta pH^2 + \left(\frac{\partial p_sK_a}{\partial \kappa}\right)^2 \Delta\kappa^2 + \left(\frac{\partial p_sK_a}{\partial C_{acid}}\right)^2 \Delta C_{acid}{}^2} \tag{S19}$$

Where Δ indicates the uncertainty in the variable. Differentiating equation 3 with respect to each variable:

$$\frac{\partial\ p_sK_a}{\partial pH} = 1 \tag{S20}$$

$$\frac{\partial\ p_sK_a}{\partial \kappa} = \frac{C_{acid}}{\ln(10)\kappa(\kappa - C_{acid})} \tag{S21}$$

$$\frac{\partial\ p_sK_a}{\partial C_{acid}} = \frac{1}{\ln(10)(C_{acid} - \kappa)} \tag{S22}$$

Additionally uncertainty analysis with respect to κ is done:

$$\Delta\kappa = \sqrt{\left(\frac{\partial\kappa}{\partial C_{\text{indicator}}}\right)^2 \Delta C_{\text{base}}^2 + \left(\frac{\partial\kappa}{\partial\delta_{\text{obs}}}\right)^2 \Delta\delta_{\text{obs}}^2 + \left(\frac{\partial\kappa}{\partial\delta_H}\right)^2 \Delta\delta_H^2 + \left(\frac{\partial\kappa}{\partial\delta_L}\right)^2 \Delta\delta_L^2} \tag{S23}$$

Differentiating equation 1 with respect to each variable yields:

$$\frac{\partial\kappa}{\partial C_{\text{indicator}}} = \frac{\delta_{\text{obs}} - \delta_L}{\delta_H - \delta_L} \tag{S24}$$

$$\frac{\partial\kappa}{\partial\delta_{\text{obs}}} = \frac{C_{\text{indicator}} \times \delta_L}{\delta_H - \delta_L} \tag{S25}$$

$$\frac{\partial\kappa}{\partial\delta_H} = \frac{C_{\text{indicator}} \times (\delta_L - \delta_{\text{obs}})}{(\delta_H - \delta_L)^2} \tag{S26}$$

$$\frac{\partial\kappa}{\partial\delta_L} = -\frac{C_{\text{indicator}} \times (\delta_H - \delta_{\text{obs}})}{(\delta_L - \delta_H)^2} \tag{S27}$$

For uncertainty estimation of fractional volume of DMSO measurement 2D ^{1}H CSI experiments were with homogenous solutions containing varied fractional volumes of DMSO/H_2O ranging from 80/20 DMSO/H_2O to 20/80 DMSO/H_2O with their fractional volume of DMSO measured for each slice of the experiment and the standard deviation calculated for all the different solvent compositions and averaged. The standard deviation obtained was ± 0.27% fractional volume of DMSO.

The uncertainty in δ_L and δ_H can be taken as 0.01 ppm (Section S11). Given the close matching of indicator and analyte pK_a values (Table S1), the dominant term in Equation S19 is the uncertainty in pH (uncertainty in indicator pK_a).[17] The uncertainty in the fitted pK_a values in Table S1 can thus be assumed equal to the uncertainty in the pK_a values of the indicators used.

S11 Fitting plots to determine pK_a , δ_H and δ_L of indicator compounds at any DMSO/H_2O solvent composition.

We note that the mole fraction of DMSO increases substantially from 0.2 at 50/50 DMSO/H_2O to 0.5 at 80/50 DMSO/H_2O. Nevertheless, this region represents only a small subset of the datapoints collected during a solvent gradient experiment (Figure S6), where the volume fraction of DMSO varies almost linearly along the tube. As such, our experimentally measured variables provide adequate coverage of the experimentally accessible range and linear Yasuda-Shedlovsky plots are obtained (Figure 4).

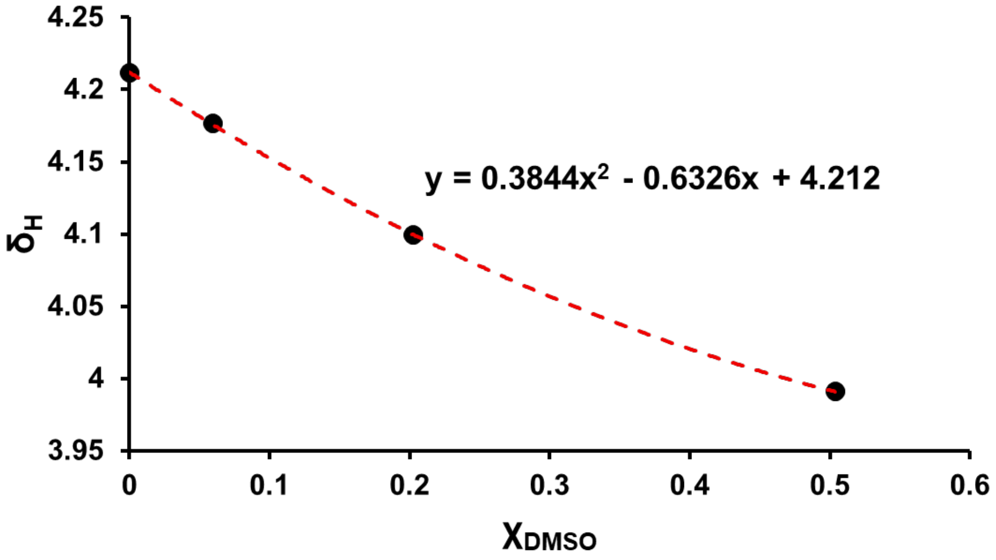

Figure S7: Plot of δ_H of glycolic acid as a function of mole fraction of DMSO with the respective fitting and equation

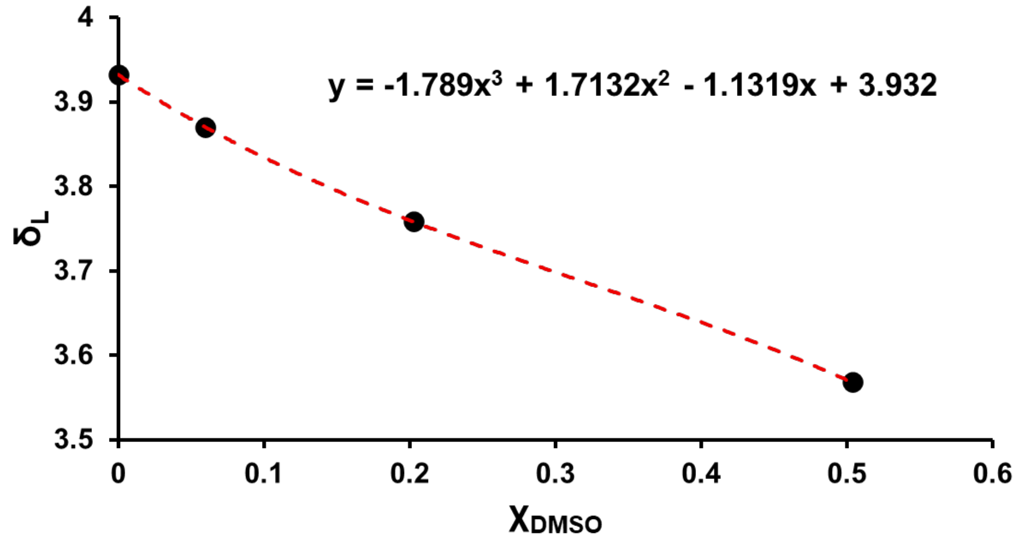

Figure S8: Plot of δ_L of glycolic acid as a function of mole fraction of DMSO with the respective fitting and equation

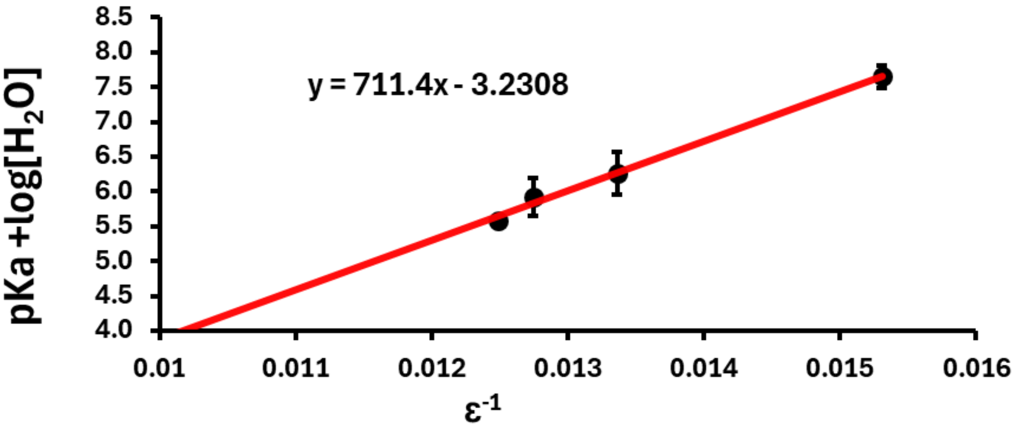

Figure S9: Plot of $log[\mathrm{H_2O}] + p_sK_a$ versus ε^{-1} of glycolic acid with respective fitting

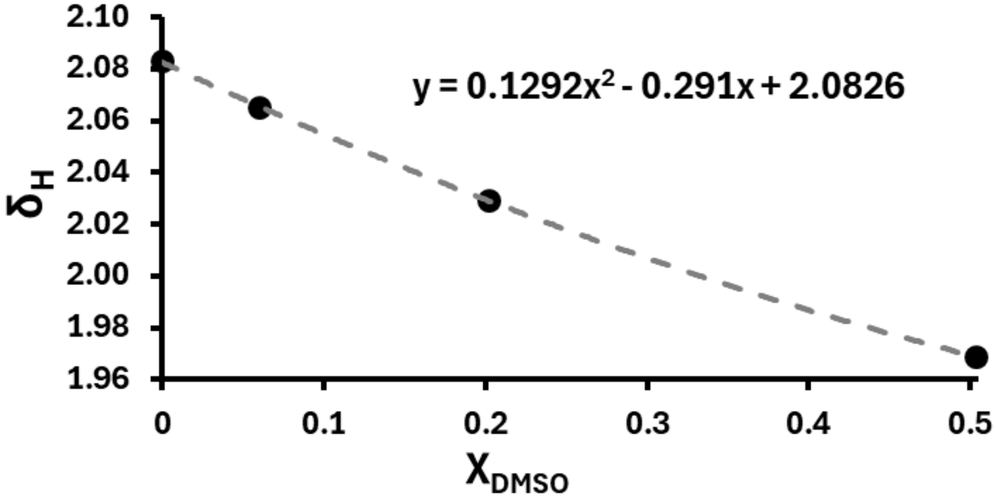

Figure S10: Plot of δ_H of acetic acid as a function of mole fraction of DMSO with the respective fitting and equation

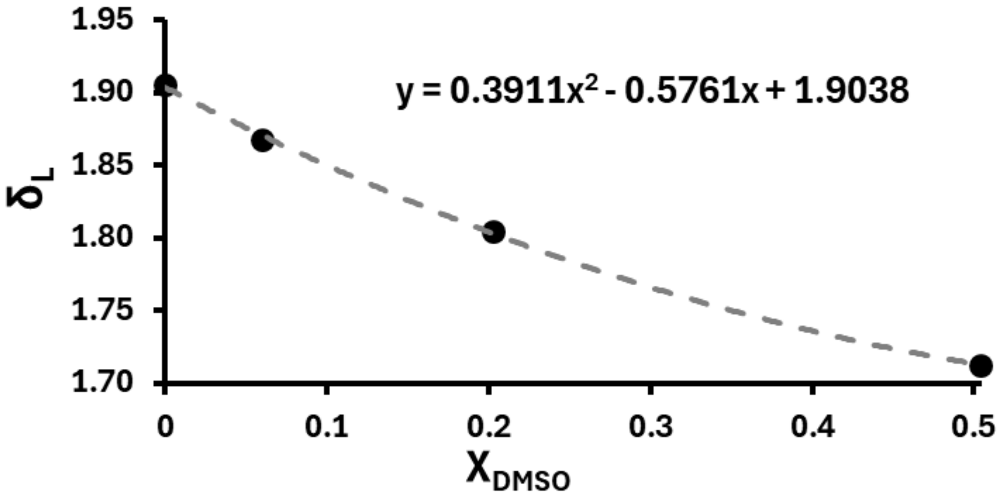

Figure S11: Plot of δ_L of acetic acid as a function of mole fraction of DMSO with the respective fitting and equation

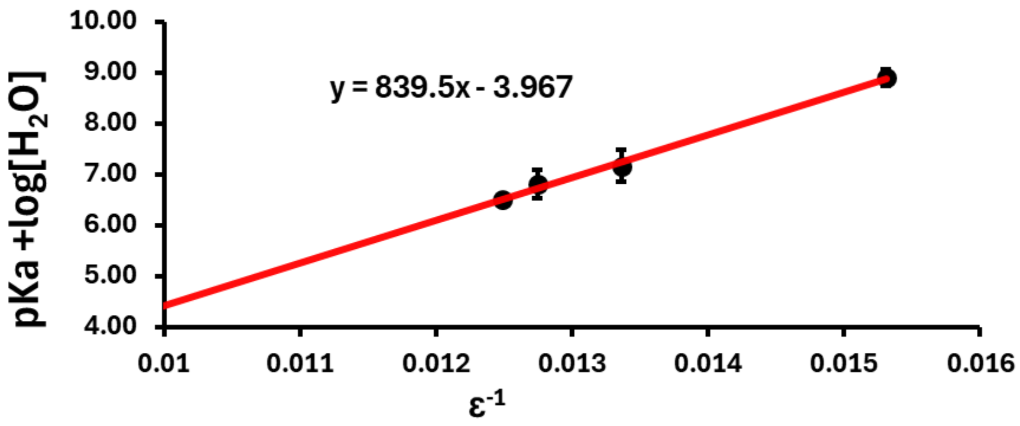

Figure S12: Plot of $log[\mathrm{H_2O}] + p_sK_a$ versus ε^{-1} of acetic acid with respective fitting

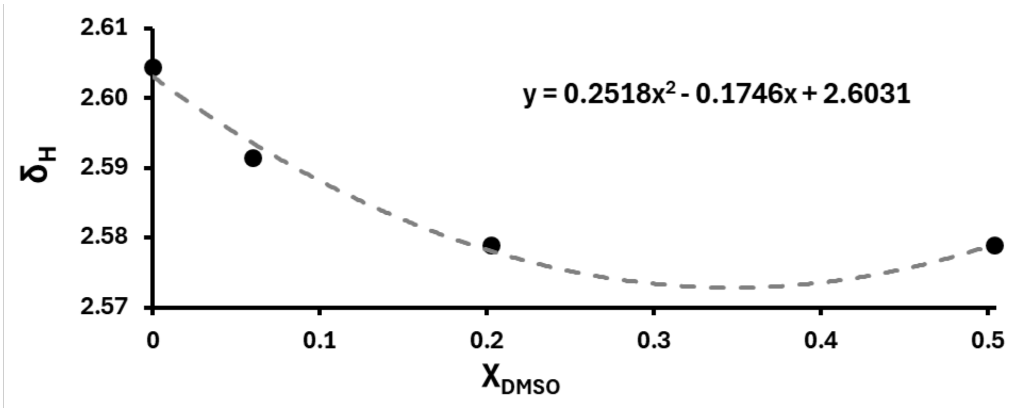

Figure S13: Plot of δ_H of 2-methylimidazole as a function of mole fraction of DMSO with the respective fitting and equation

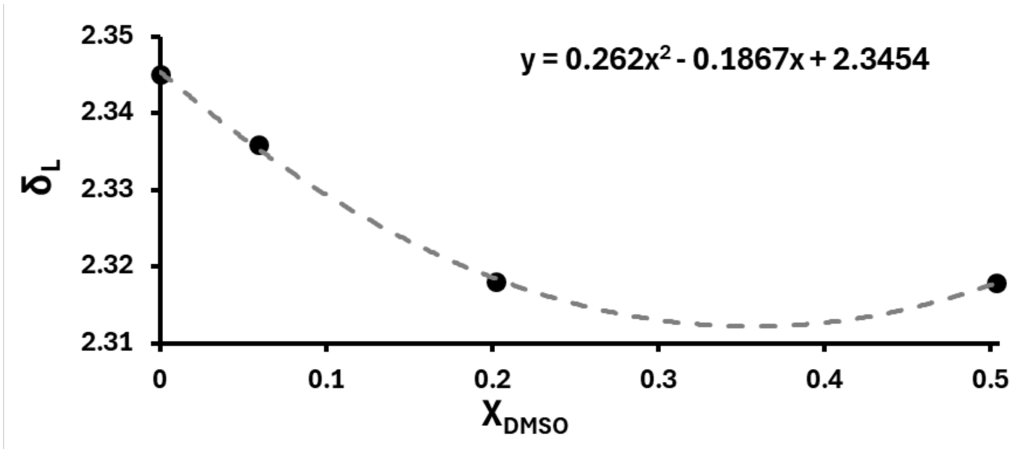

Figure S14: Plot of δ_L of 2-methylimidazole as a function of mole fraction of DMSO with the respective fitting and equation

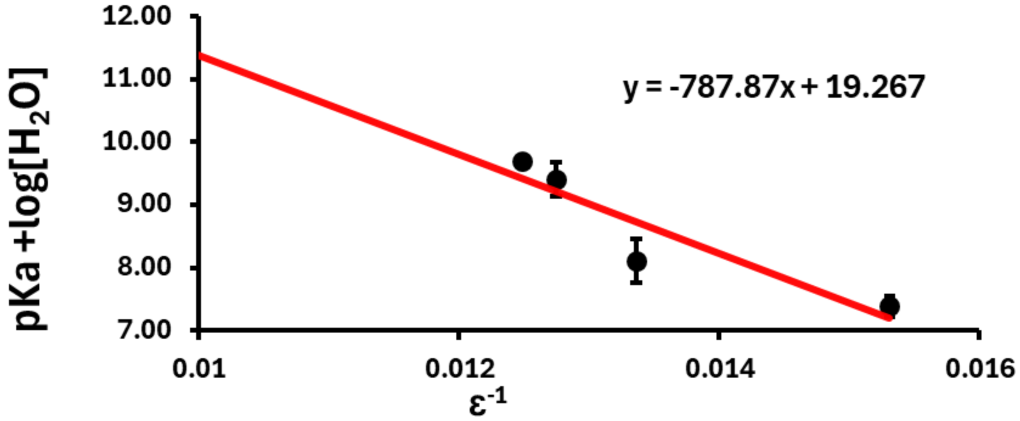

Figure S15: Plot of $log[\mathrm{H_2O}] + p_sK_a$ versus ε^{-1} of 2-methylimidazole with respective fitting

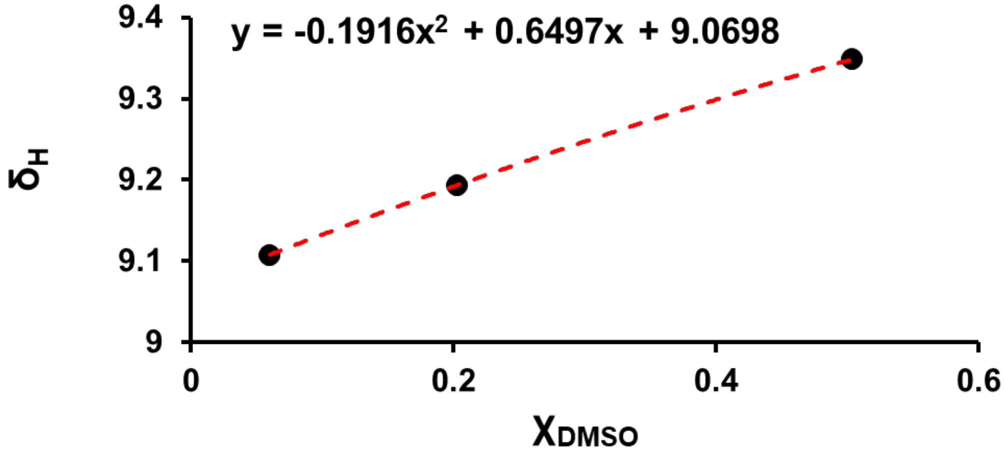

Figure S16: Plot of δ_H of 1,2,4-triazole as a function of mole fraction of DMSO with the respective fitting and equation

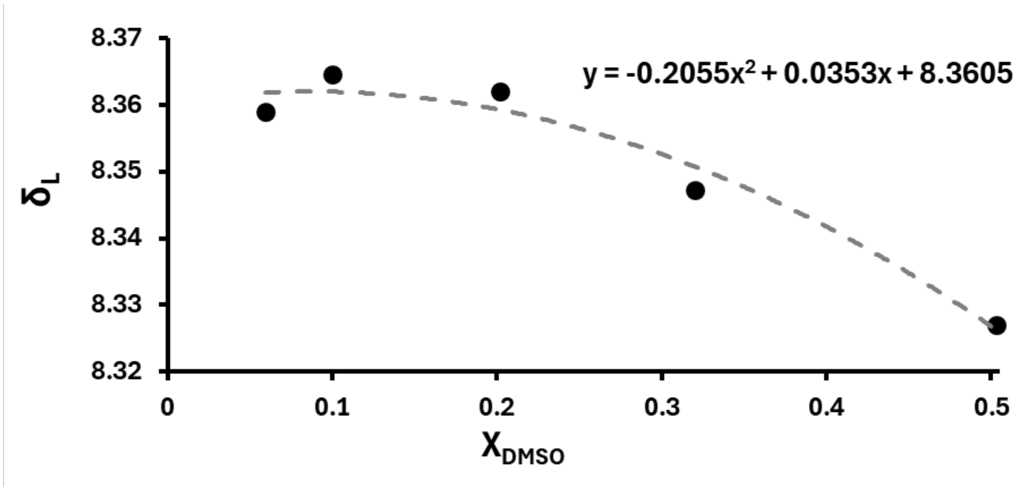

Figure S17: Plot of δ_L of 1,2,4-triazole as a function of mole fraction of DMSO with the respective fitting and equation

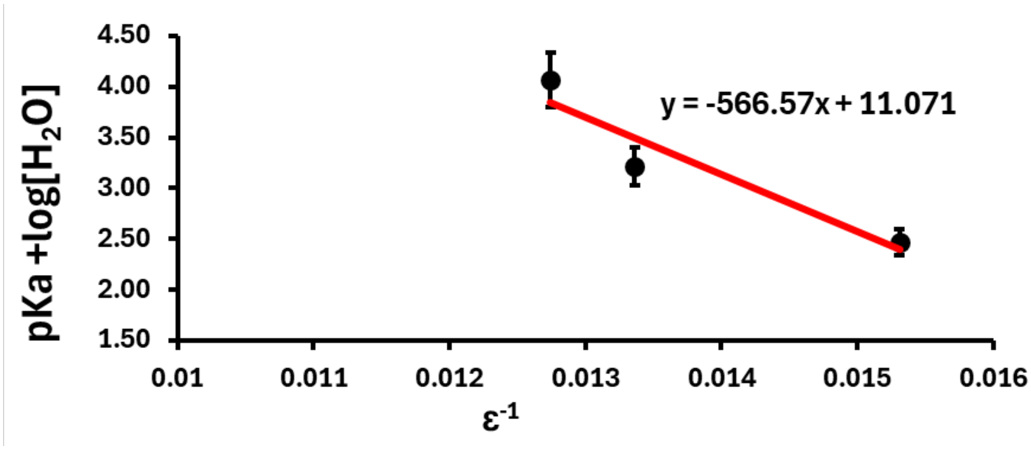

Figure S18: Plot of $log[H_2O] + p_sK_a$ versus ε^{-1} of 1,2,4-triazole with respective fitting

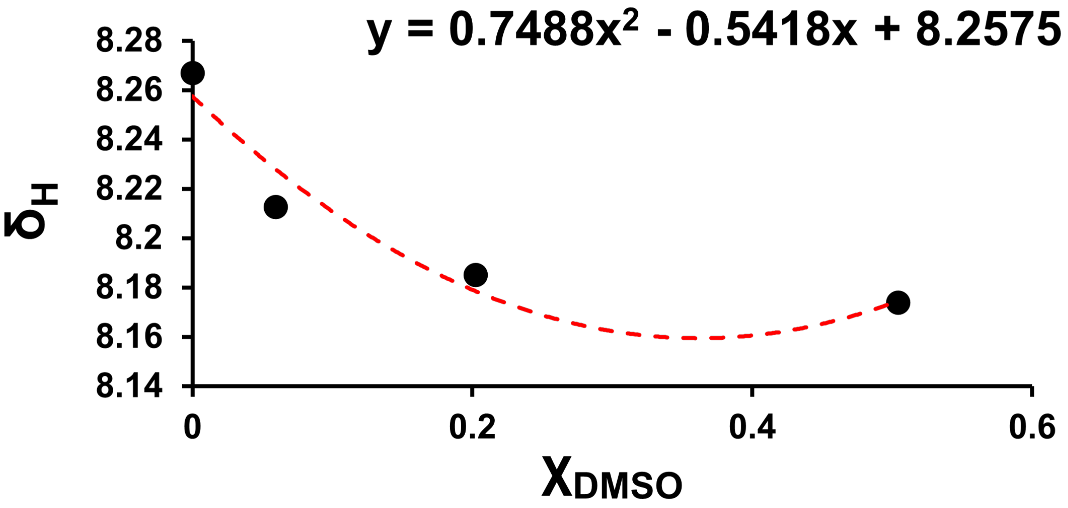

c

Figure S19: Plot of δ_H of sodium formate as a function of mole fraction of DMSO with the respective fitting and equation

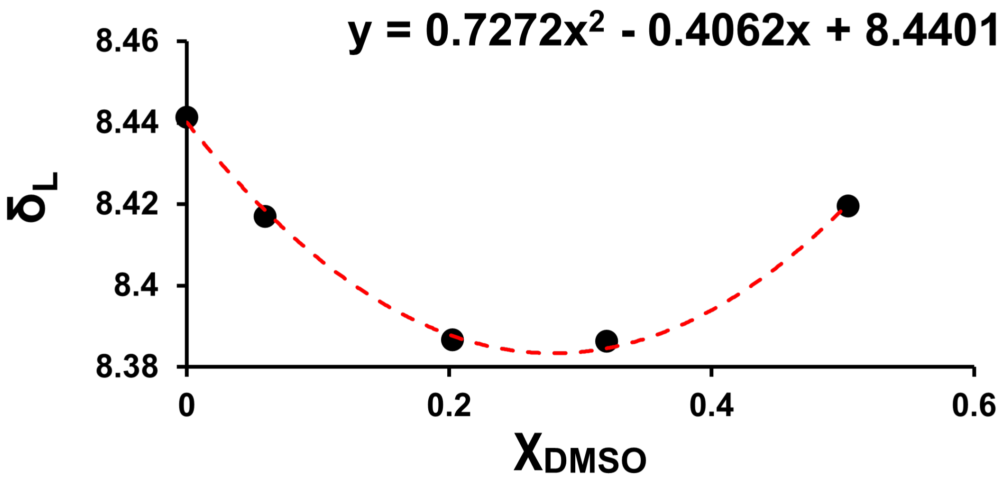

Figure S20: Plot of δ_L of sodium formate as a function of mole fraction of DMSO with the respective fitting and equation

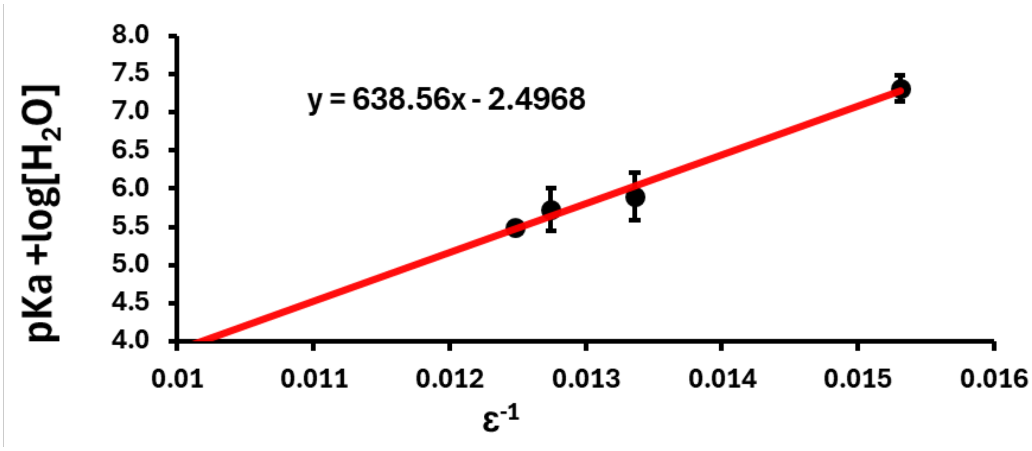

Figure S21: Plot of $log[\mathrm{H_2O}] + p_sK_a$ versus ε^{-1} of sodium formate with respective fitting

S12 Creation of solvent composition gradients between DMSO and $CHCl_3$ and analysis by high-field NMR

As for the other solvent systems presented in the manuscript, samples are prepared based on the contrasting density and miscibility of the two solvents. The NMR tube was filled to 20 mm height with a solution of hexamethyldisilane (0.7 vol%) and tert-butanol (10 mM) in $CHCl_3$. A solution of the same solutes in DMSO-d_6 was then pipetted on top to a total height of 40 mm and the sample stood for 7 hours before analysis. The ^{1}H chemical shift of $CHCl_3$ referenced to HMDS can be fitted to a third order polynomial that relates vol% $CHCl_3$ to the observed chemical shift (Figure S22b). Separate resonances for the water and *tert*-butanol can be observed below 30 vol% $CHCl_3$, in common with the observations of Gottlieb et al. [18], suggesting that these gradients could be used to study effects such as intramolecular hydrogen bonding. [19]

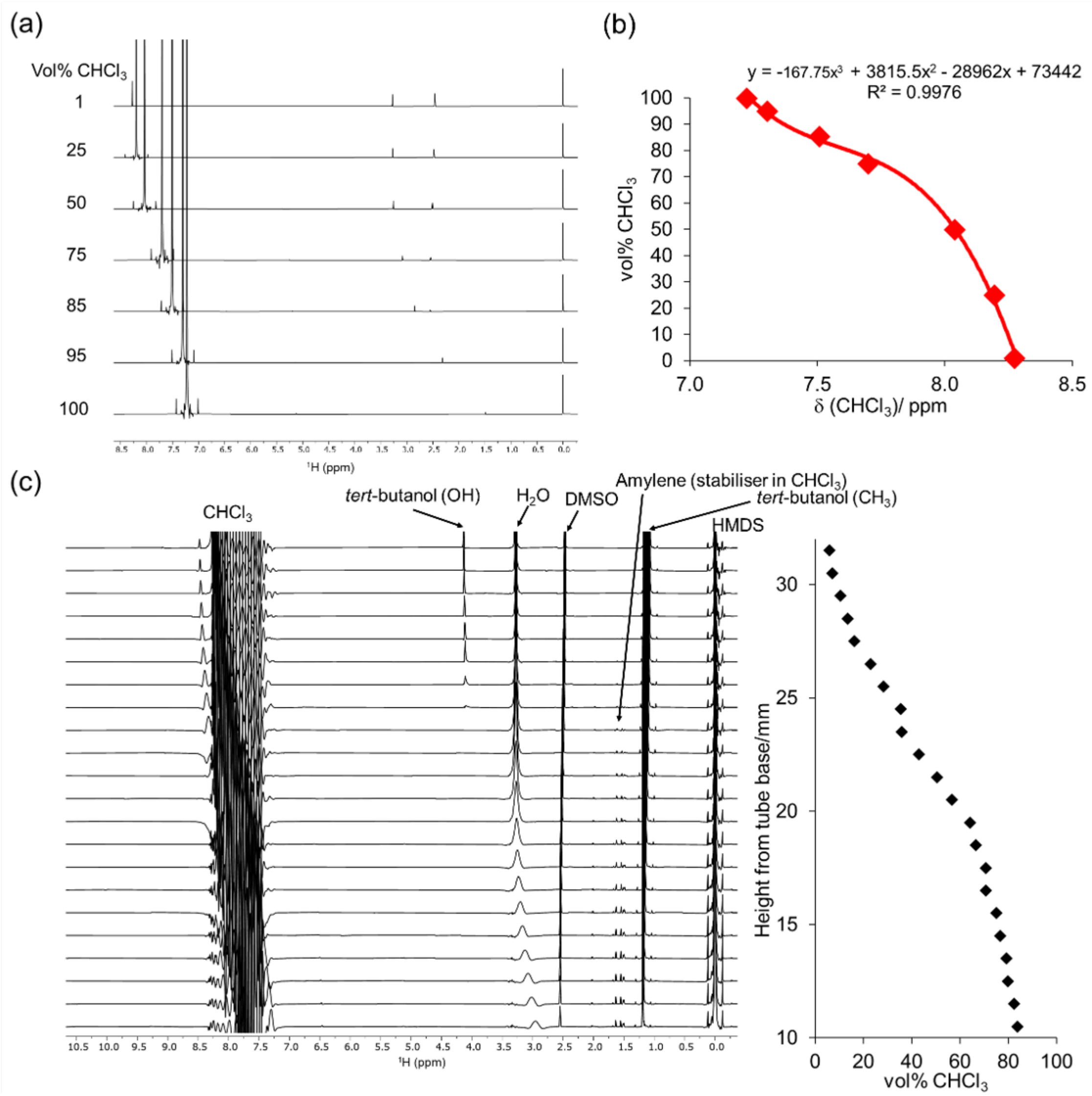

Figure S22: (a) 1D Calibration spectra showing shift of $CHCl_3$ resonance in DMSO/$CHCl_3$ as proportion of $CHCl_3$ is increased. (b) Fit of ^{1}H chemical shift of $CHCl_3$ to polynomial function to enable determination of solvent composition along gradient. (C) CSI dataset recorded along a DMSO/$CHCl_3$ composition gradient. The solvent composition at each height from the tube base is indicated and aligned with the corresponding spectra in the CSI dataset.

References

[1] H. Hussain, Y. Z. Khimyak and M. Wallace, *Anal. Chem.*, 2024, **96**, 19858–19862.

[2] M. Wallace, N. Abiama and M. Chipembere, *Anal. Chem.*, 2023, **95**, 15628–15635.

[3] R. Yang and S. G. Schulman, *Talanta*, 2003, **60**, 535–542.

[4] J. Mollin, Z. Pavelek, J. Navratilova and A. Recmanova, *Collect. Czechoslov. Chem. Commun.*, 1985, **50**, 2670–2678.

[5] P. De Maria, A. Fini, A. Guarnieri and L. Varoli, *Archiv der Pharmazie*, 1983, **316**, 559–563.

[6] R. Eliason and M. Kreevoy, *J. Phys. Chem.*, 1974, **78**, 2658–2659.

[7] N. McHedlov-Petrossyan and R. Mayorga, *J. Chem. Soc. Faraday Trans.*, 1992, **88**, 3025–3032.

[8] E. Baughman and M. Kreevoy, *J. Phys. Chem.*, 1974, **78**, 421–423.

[9] S. Ledenkov, V. Sharnin and V. Isaeva, *Zhurnal Fizicheskoi Khimii*, 1995, **69**, 994–996.

[10] A. Jouyban, S. Soltanpour and H.-K. Chan, *Int. J. Pharm.*, 2004, **269**, 353–360.

[11] I. Płowaś, J. Świergiel and J. Jadżyn, *J. Chem. Eng. Data*, 2013, **58**, 1741–1746.

[12] W. Yao, Z. Yu, S. Wen, H. Ni, N. Ullah, Y. Lan and Y. Lu, *Chem. Sci.*, 2017, **8**, 5196–5200.

[13] I. G. Egorov and D. M. Makarov, *Russ. J. Phys. Chem. A*, 2009, **83**, 693 – 698.

[14] J. Crank, *The Mathematics of Diffusion*, Clarendon Press, 1979.

[15] K. J. Packer and D. J. Tomlinson, *Trans. Faraday Soc.*, 1971, **67**, 1302–1314.

[16] D. L. Gurina and V. A. Golubev, *Results Chem.*, 2022, **4**, 100673.

[17] M. Wallace, K. Lam, A. Kuraite and Y. Z. Khimyak, *Anal. Chem.*, 2020, **92**, 12789–12794.

[18] H. E. Gottlieb, V. Kotlyar and A. Nudelman, *J. Org. Chem.*, 1997, **62**, 7512–7515.

[19] M. H. Abraham, R. J. Abraham, A. Aghamohammadi, K. Afarinkia and X. Liu, *J. Mol. Liq.*, 2020, **315**, 113730.
